# Pulmonary fibroblast subsets demonstrate differentially enriched signaling pathways during fibrosis resolution and repair

**DOI:** 10.1172/jci.insight.199660

**Published:** 2026-05-05

**Authors:** Daniel G. Foster, Nomin Javkhlan, Bart P. Black, Brian E. Vestal, David W.H. Riches, Elizabeth F. Redente

**Affiliations:** 1 Department of Pharmaceutical Sciences, University of Colorado School of Pharmacy, Aurora, Colorado, USA.; 2 Department of Pediatrics, and; 3 Center for Genes, Environment and Health, National Jewish Health, Denver, Colorado, USA.; 4 Division of Pulmonary, Allergy and Critical Care Medicine, Department of Medicine, and; 5 Department of Immunology and Microbiology, University of Colorado Anschutz Medical Campus, Aurora, Colorado, USA.; 6 Department of Research, Veterans Affairs Eastern Colorado Health Care System, Aurora, Colorado, USA.

**Keywords:** Cell biology, Pulmonology, Fibrosis, Molecular genetics

## Abstract

The lungs have a remarkable capacity to undergo homoeostatic repair and regeneration after injury, which often occurs in patients with acute respiratory distress syndrome (ARDS) and in the single-dose bleomycin mouse model. Fibroblasts are critical mediators of fibrotic disease and RNA sequencing has identified significant heterogeneity within pulmonary fibroblast populations. However, the contribution of distinct fibroblast subsets to the repair process has been understudied compared with their role in fibrosis initiation and progression. Therefore, we sought to define the transcriptional landscape of 3 phenotypically defined fibroblast subsets that occupy discrete spatial locations in naive lungs. Using TdTomato-lineage tracing approaches, we identified and interrogated collagen1a1^+^ (*Col1a1*) fibroblasts, perilipin 2^+^ (*Plin2*) alveolar fibroblasts, and α-smooth muscle actin^+^ (*Acta2*) myofibroblasts during fibrosis development and resolution after single-dose bleomycin. Quantification of fibroblast numbers showed that all 3 subsets expanded during fibrosis and contracted toward naive levels with resolution. Principal component and gene set enrichment analyses indicate that each subset underwent major transcriptomic shifts during fibrosis development, converging on a similar profibrotic transcriptional profile. However, during resolution, *Plin2^+^* and *Acta2^+^* fibroblasts reverted toward a prefibrotic transcriptional state, whereas *Col1a1*^+^ fibroblasts acquired a distinct program that suggests an active role in mediating the repair processes.

## Abstract 2

New research shows how specific lung fibroblast subsets can drive repair after fibrotic lung injury and identified a potential unique Col1a1+ cells in fibrosis resolution.N/A

## Graphical abstract

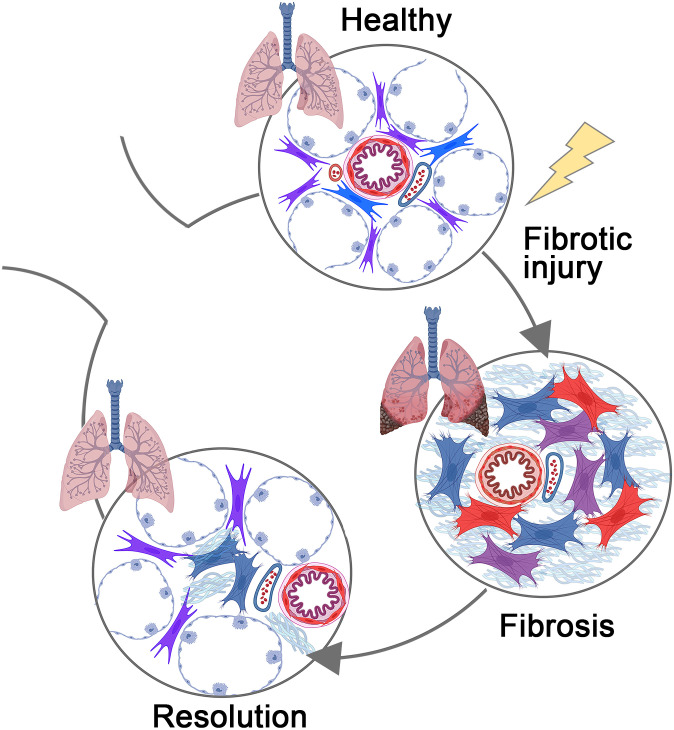

## Introduction

The single-dose bleomycin model of acute lung injury (ALI) and subsequent fibrosis development is the most widely used and well-characterized animal model of transient resolving pulmonary fibrosis in mice. It undergoes spontaneous resolution of diffuse alveolar damage and fibrosis with lung architecture and collagen levels returning to preinjury levels in young mice (1–3). This model recapitulates the disease course of many patients with acute respiratory distress syndrome (ARDS) where lung biopsies show histopathological evidence of fibroproliferation in the majority of patients with ARDS (4–6). In the acute setting, significant increases in procollagen I and III can be measured in the bronchoalveolar lavage fluid (BALF) of patients as early as 72 hours after disease onset, and elevated levels of procollagen have been shown to correlate with increased mortality (7, 8). Long-term follow-up studies from 6 months to 5 years after hospital discharge show that most ARDS survivors recover much of their lung function together with diminished fibrosis-associated reticular infiltrates and bronchiectasis (9–11). This clinical evidence suggests that fibroproliferative injury and collagen accumulation in the human lung can be a self-limiting and resolving event. The bleomycin model recapitulates this disease course and reproducibly demonstrates the capacity of the lungs to repair after a significant injury and fibrotic response (12). However, the resolution phase and the resulting repaired lung have historically been understudied compared with the injury and fibrosis phases. The pathobiology of lung fibrosis has long been described as maladaptive wound healing, and fibroblasts are central regulators in both pulmonary fibrosis and homeostatic wound repair (13–16). A better understanding of how the transcriptional profiles of multiple fibroblast subsets respond during fibrosis and subsequent resolution is an important area of study that may lead to the identification of new targets and strategies for therapeutic intervention. We and others have shown that mesenchymal fibroblasts have increased expression of profibrotic and extracellular matrix (ECM) genes during the peak of the fibrotic response after single-dose bleomycin (16–19). During resolution, the fibroblast transcriptomic signature changes and is associated with wound healing terms and reduction of profibrotic terms by 6 weeks after bleomycin (16, 19). However, a thorough examination of the transcriptional profile of fibroblast subsets during fibrosis resolution has yet to be conducted.

There is considerable heterogeneity in the profibrotic fibroblast populations, with many of these cells having high expression of *Col1a1*, including interstitial, adventitial and alveolar fibroblasts, pericytes, and mesothelial cells during the development of pulmonary fibrosis (16, 17, 20–23). Previous studies have shown that Col1a1-GFP–expressing fibroblasts expand and contract following bleomycin injury and have significantly different transcriptional profiles at day 14 and 30, coinciding with peak fibrosis and early resolution (16, 17, 19). Lineage labeling of *Acta2^+^* myofibroblasts and adipocyte differentiation–related protein–expressing (ADRP/*Plin2^+^*) alveolar fibroblasts also demonstrated this complexity of cross-phenotypes by undergoing a lipogenic to myogenic conversion during bleomycin-induced fibrosis development and a reversal of this phenotype during fibrosis resolution (24). However, little is known about if and/or how these distinct fibroblast populations directly contribute to fibrosis resolution.

To address this knowledge gap, there are complementary experimental strategies that could be leveraged to identify and follow fibroblast subset during the trajectory of fibrosis development and resolution, as shown in Supplemental Figure 1 (supplemental material available online with this article; https://doi.org/10.1172/jci.insight.199660DS1). First, gene reporter mice including Col1a1-GFP and PDGFRα-GFP mice enable fibroblast gene expression to be followed in real time (19, 25). Second, inducible Cre drivers with tamoxifen delivery prior to injury can be used for classical lineage tracing of initial naive populations (17, 23, 24, 26–28), which follows a lineage regardless of whether it upregulates fibrotic genes, participates in fibrosis, or facilitates fibrosis resolution. Third, inducible Cre drivers with tamoxifen delivery after injury have been used as a strategy to capture subsets as they expand under common fibrosis-associated drivers during disease progression and which allows tracking of daughter cells that participated in fibrosis into the resolution time points (16) (Supplemental Figure 1). Each strategy has advantages and disadvantages in analyzing transcriptional changes in fibroblast subsets. In a complex disease state like fibrosis, analyzing fibroblast subsets via all 3 strategies is important to identifying global changes and patterns that one strategy alone is insufficient to capture (Supplemental Figure 1).

This is relevant to interventional studies interested in using Cre drivers to manipulate fibroblast functions following lung injury. We sought to address this gap by determining transcriptional profile changes within fibroblast subsets targeted by all 3 common inducible Cre drivers (*Col1a1*, *Acta2*, and *Plin2*), with labeling throughout the fibroproliferative expansion phase of fibrosis and assessment during active fibrosis and fibrosis resolution. This complex labeling strategy captures both lineage (early tamoxifen injections) and subsets as they gain known fibrotic gene signatures (later tamoxifen injections, specifically with the *Col1a1* and *Acta2* transgenic lines). Our findings highlight the convergence and overlap of multiple subsets that become involved during fibrosis as well as their flexibility to return to distinct transcriptional profiles after disease resolution.

## Results

### Col1a1^+^, Plin2^+^, and Acta2^+^ pulmonary fibroblast subsets expand during bleomycin-induced fibrosis and contract during resolution.

**Figure 1:**
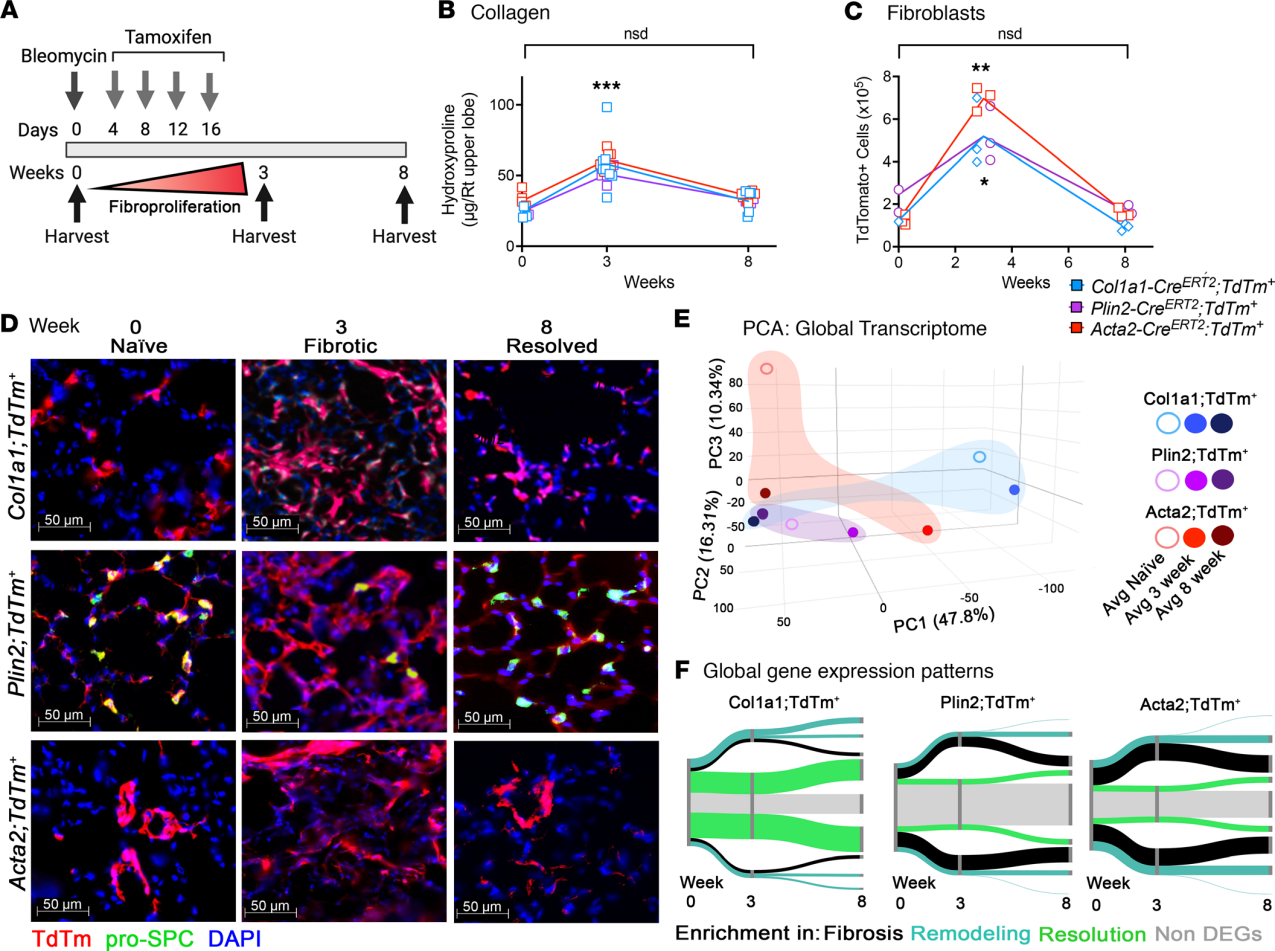
Characterization of fibroblast subsets during fibrosis and spontaneous resolution. (**A**) Schematic of in vivo tamoxifen treatment and harvest times after bleomycin. (**B**) Total lung collagen levels quantified by hydroxyproline in the lungs over time after bleomycin, *n* = 5 mice/group. (**C**) Flow cytometry quantification of TdTm^+^ fibroblasts in each subset over time, *n* = mice/group. (**D**) Representative 20× fluorescence images from fixed frozen lung of naive, 3-week and 8-week lungs. TdTomato (red); pro-SPC^+^ AT2 cells (green); nuclei (DAPI, blue). (**E**) Principal-Component Analysis (PCA) plots of bulk RNA-seq subsets over time. (*Col1a1*^+^, blue; *Plin2*^+^, purple; *Acta2*^+^, red; naive, open circles; 3 weeks, bright circles; 8 weeks, dark circles). (**F**) Sankey plots categorizing differentially expressed genes (DEGs) across time into fibrosis-associated (black), resolution-associated (green), and remodeling-associated (teal) trajectories. Data are shown as mean ± SEM. **P < 0.01, ***P < 0.001, 3-week bleomycin compared with 0 and 8 weeks by 2-way ANOVA between all 3 subset samples.

In order to define the transcriptional landscape of fibroblast subsets during homeostasis, fibrosis and resolution, we labeled *Col1a1*^+^, *Plin2*^+^, and *Acta2*^+^ fibroblast subsets with TdTomato (TdTm^+^) at time 0 for controls, or beginning 4 days after single-dose bleomycin injury during the fibroproliferative phase to capture fibroblast subsets that are actively expressing these subset markers during fibrosis development, to account for their phenotypic plasticity during this time (Figure 1A). At peak fibrosis 3 weeks after bleomycin injury, there was a significant increase in total lung collagen content by hydroxyproline assay and increased numbers of TdTm^+^ fibroblasts (lineage negative: CD45^–^, EpCAM^–^, CD31^–^) by flow cytometry in all subsets (Figure 1, B and C). By 8 weeks, spontaneous resolution occurred, as demonstrated by near-normal histology and a return of hydroxyproline collagen levels and TdTm^+^ fibroblast numbers to naive levels in all subsets (Figure 1, B and C) (16). Fluorescence imaging of TdTm^+^ cells in naive lung sections showed *Col1a1*^+^ matrix–producing fibroblasts within the alveolar interstitium; *Plin2*^+^ alveolar fibroblasts located within the parenchyma adjacent to proSPC^+^ alveolar type 2 (AT2) cells, which also express *Plin2*; and *Acta2^+^* cells surrounding the vasculature and large airways (Figure 1D). At peak fibrosis, all 3 TdTm^+^ subsets were present within fibrotic areas, but after resolution, their localization was similar to that of the naive lung (Figure 1D).

PCA plots of bulk RNA-seq from isolated TdTm^+^ fibroblasts indicate significant global transcriptional changes in all 3 populations at all timepoints (Figure 1E and Supplemental Figure 2). In naive lungs, *Col1a1^+^*, *Plin2^+^*, and *Acta2^+^* subsets showed large differences in their transcriptomes, as would be expected for distinct fibroblast subsets. The *Col1a1^+^* subset had 5,271 differentially expressed genes (DEGs), the *Plin2^+^* subset had 892 DEGs, and the *Acta2^+^* subset had 1,729 DEGs. Gene set enrichment analysis (GSEA) was performed on these gene sets, and uniquely enriched biological processes were identified using the Gene Ontology (GO) database resulting in 517, 131, and 106 significantly enriched Gene Ontology:Biological Process (GO:BP) terms in *Col1a1*^+^, *Plin2*^+^, and *Acta2^+^* fibroblast subsets, respectively. In the *Col1a1^+^* subset, enriched pathways were associated with Wnt signaling and regulation of ECM and TGF-β receptor signaling. This suggests that, at baseline, these cells may play a central role in processes associated in collagen fibril organization, cellular response to growth factors, and regulation of epithelial cell populations through managing their migration and proliferation (Supplemental Figure 3A). *Plin2^+^* fibroblasts were enriched for genes associated with lipid regulation and metabolic control, along with autophagy and organelle communication and terms associated with regulation of alveolar niche cell growth and tissue architecture (Supplemental Figure 3B). At baseline, the *Acta2^+^* subset was mostly composed of smooth muscle cells, and its transcriptomic signature was differentially enriched for pathways associated with immune cell regulation, chemotaxis, and migration along with the positive regulation of Erk1 and Erk2, which is a critical pathway for cell motility and contraction regulated by *Acta2* (Supplemental Figure 3C). We also examined the 2,595 genes that were differentially expressed between all 3 subsets at baseline. The 153 GO:BP pathways were associated with vascular development (angiogenesis, endothelial cell sprouting, and endothelial cell barrier function) along with pathways linked to TGF-β and VEGF signaling (Supplemental Figure 3D).

We also observed a significant transcriptional shift during fibrosis, with all subsets displaying transcriptional similarity related to fibrosis-associated pathways (Figure 1E and Supplemental Figure 2). Interestingly, after 8 weeks, the global transcriptional profile of all subsets converged and were transcriptionally more similar to each other than in their naive states (Figure 1E and Supplemental Figure 2). However, while the *Plin2^+^* and *Acta2^+^* population transcription profiles moved toward their respective naive states, the *Col1a1^+^* fibroblasts moved farther away from their naive profile, maintaining a significantly different transcriptomic profile after fibrosis resolution (Figure 1E and Supplemental Figure 2).

### Defining global DEG transcriptional patterns during fibrosis resolution and repair.

Sankey plots were generated to define a more granular description of the global patterns of DEGs over the 8-week time course and to organize the data into 3 broad gene expression profile patterns: (a) fibrosis-associated, (b) remodeling-associated, and (c) resolution-associated (Figure 1F). Fibrosis-associated DEGs were defined as genes that were significantly different at 3 weeks after bleomycin compared with naive and then returned to naive levels by 8 weeks. Fibrosis-associated DEGs were highly enriched in *Plin2*^+^ and *Acta2*^+^ fibroblasts, respectively, encompassing 55.13% (4,037 genes/total DEGs) and 54.15% (5,282 genes/total DEGs) of DEGs across the time course, versus only 10.29% (1,102 genes/total DEGs) for *Col1a1*^+^ fibroblasts (Figure 1F). Remodeling-associated genes were defined as genes with a significant differential expression at 3 weeks compared with naive that continued to have significant differential expression at 8 weeks compared with naive. These genes contained a similar portion of total DEGs across all 3 subsets, with 20.47% (2,192 genes/total DEGs), 20.84% (1,526 genes/total DEGs), and 27.07% (2,641 genes/total DEGs) in *Col1a1^+^*, *Plin2^+^*, and *Acta2^+^* fibroblasts, respectively (Figure 1F, teal lines). Resolution-associated DEGs were defined as genes that did not change at 3 weeks compared with naive but showed significant differential expression (up or down) at 8 weeks compared with naive. These were highly enriched in *Col1a1^+^* fibroblasts encompassing 69.24% (7,414 genes/total DEGs) of DEGs across the time course compared with only 24.03% (1,760 genes/total DEGs) and 18.78% (1,832 genes/total DEGs) for *Plin2^+^* and *Acta2^+^* fibroblasts, respectively (Figure 1F). The large differences in the fibrosis- and resolution-associated gene expression patterns between the *Col1a1^+^* fibroblasts and the other 2 subsets explains the difference in the transcriptional profiles between subsets in the global PCA plot (Figure 1E). Additionally, it suggests that *Col1a1^+^* fibroblasts have: (a) persistent reprogramming from the naive state even after fibrosis resolution and (b) a distinct transcriptional program during resolution that is associated with repair in addition to extinguishing profibrotic gene expression, a gene expression profile that was not observed in the *Plin2^+^* and *Acta2^+^* subsets.

### Defining fibrosis-associated DEG transcriptional patterns in fibroblast subsets.

**Figure 2:**
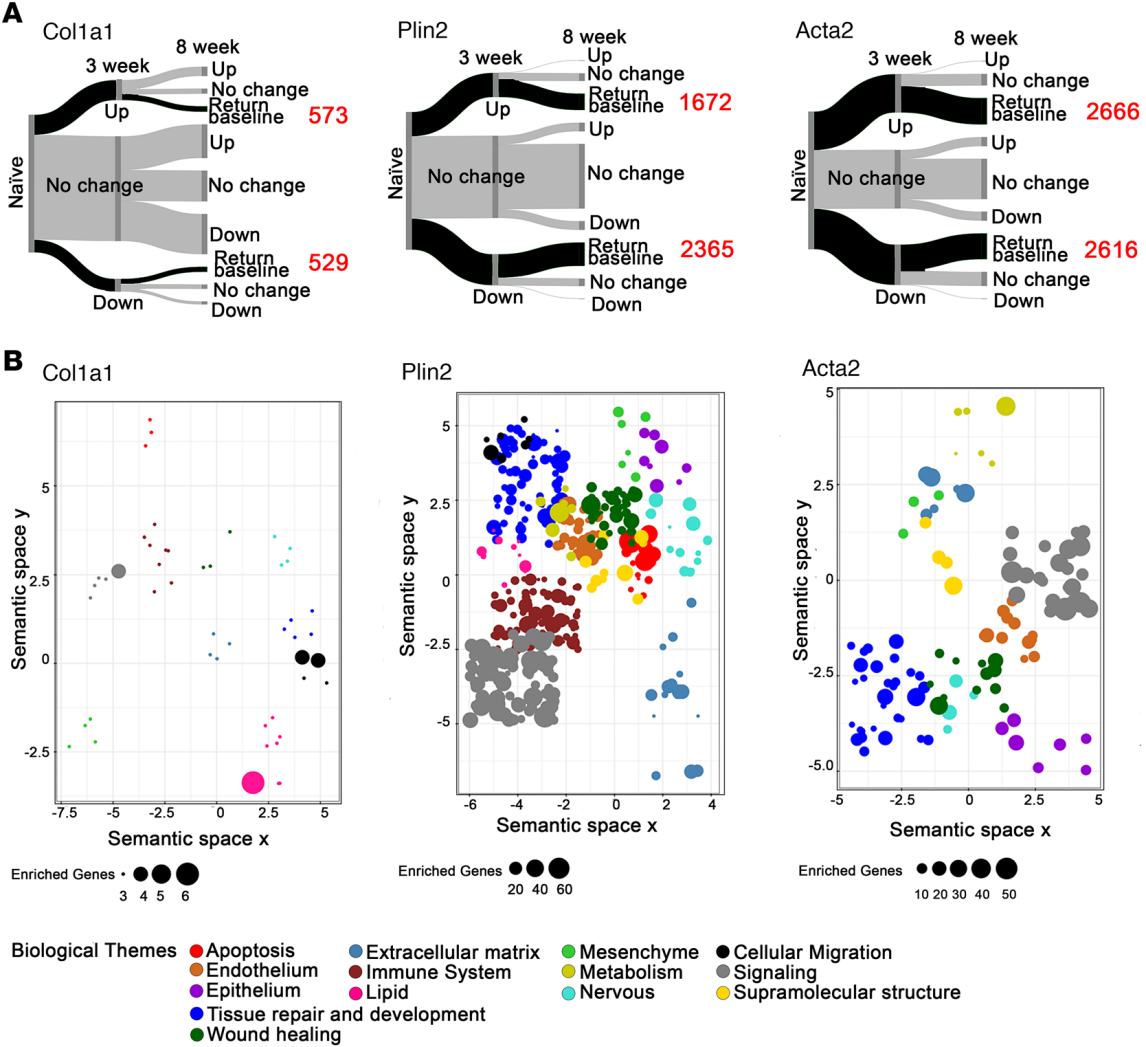
Fibrosis-associated genes and biological pathways are enriched in the *Plin2*^+^ and *Acta2*^+^ subsets at 3 weeks. (**A**) Sankey plots for fibrosis-associated DEG transcriptional programing in black, across subsets. Fibrosis-associated DEGs are genes significantly altered at 3 weeks versus naive (FDR ≤ 0.05) and return to baseline by 8 weeks versus naive (FDR ≥ 0.05). (**B**) Semantic-similarity clustering of significantly enriched Gene Ontology Biological Process (GO:BP) terms (adjusted *P* ≤ 0.05) derived from highly changed fibrosis-associated DEGs (FDR ≤ 0.05, |log_2_FC| ≥ 1) identified 14 semantic similarity clusters (SSC). Dots are individual GO:BP terms; dot size reflects the number of genes in the term; color indicates SSC.

**Figure 3:**
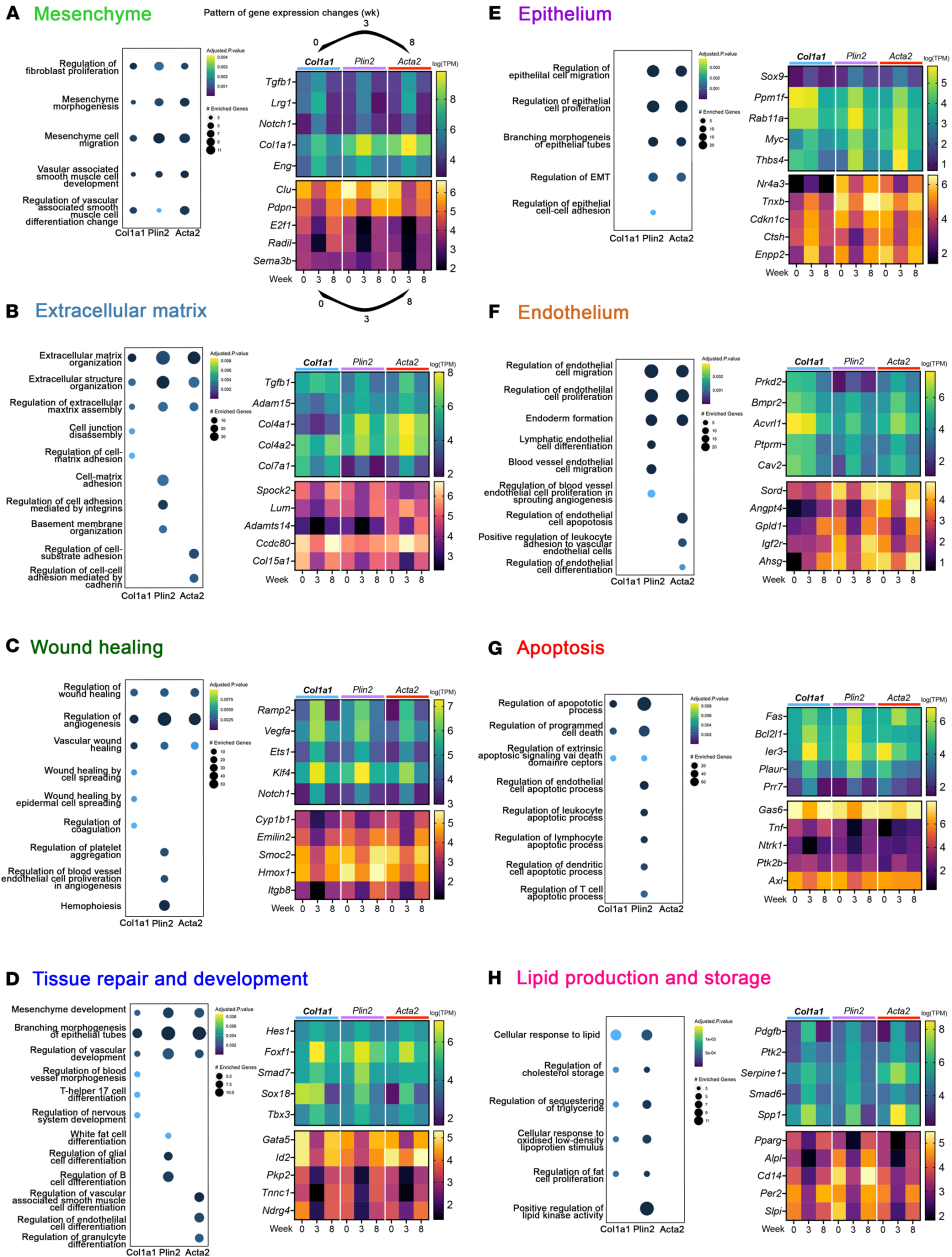
Transcriptional programming of fibrosis-associated genes are enriched in the *Plin2*^+^ and *Acta2*^+^ subsets at 3 weeks. (**A**–**H**) Dot-plots summarizing representative enriched GO:BP terms and top 5 up- and downregulated genes (log[TPM]) over the time course for selected SSCs based on changes in the *Col1a1^+^* subset: (**A**) mesenchyme; (**B**) extracellular matrix; (**C**) wound healing; (**D**) tissue repair and development; (**E**) epithelial regulation; (**F**) endothelial regulation; (**G**) apoptosis; and (**H**) lipid production and storage. Dot size indicates gene count; color indicates adjusted *P* value. Black schematic over heatmap in **A** indicates expected pattern of gene changes over the time course from the Sankey plot.

Next, we examined the gene expression profile and associated functional pathways of the fibrosis-associated DEGs in the 3 fibroblast subsets (Figure 2A). Initially, 1,102 fibrosis-associated DEGs were identified for the *Col1a1^+^* subset, 4,037 for the *Plin2^+^*, and 5,282 for the *Acta2^+^*. These were further narrowed down to only those genes that were significantly changed (|logFC| ≥ 1) at 3 weeks, which resulted in 146, 1,314, and 1,129 genes for analysis in the *Col1a1^+^*, *Plin2^+^*, and *Acta2^+^* fibroblast subsets, respectively (Supplemental Figure 4). GSEA was performed on these gene sets, and enriched biological processes were identified using the GO database resulting in 54, 441, and 143 significantly enriched GO:BP terms in *Col1a1^+^*, *Plin2^+^*, and *Acta2^+^* fibroblast subsets, respectively. Given the large number of significantly enriched gene GO:BP terms that were identified, enrichment results were further organized and consolidated into 14 biological themes using semantic similarity clustering (SSC) across the fibrosis-associated DEGs: apoptosis, endothelium, epithelium, ECM, immune system, lipid production and storage, mesenchyme, metabolism, nervous system, cellular migration, signaling, supramolecular structure, tissue repair and development, and wound healing (Figure 2B). SSCs that were enriched across all 3 subsets included biological processes associated with mesenchyme, ECM, wound healing, and tissue repair and development (Figure 2B). Within 8 of the SSCs, we determined the significantly expressed GO:BP pathways and the top 5 upregulated and downregulated DEGs across the subsets. All 3 subsets had significant enrichment in mesenchyme-associated GO:BP terms, including: regulation of fibroblast proliferation, mesenchyme morphogenesis and cell migration, and vascular associated smooth muscle cell development (Figure 3A). The most significantly increased genes associated with these biological processes across the subsets, including *Tgfb1*, *Lrg1*, *Notch1*, *Col1a1*, and *Eng*, while *Clu*, *Pdpn*, *E2f1*, *Radil*, and *Sema3b* represented the most significantly decreased genes (Figure 3A). GO:BP terms enriched under ECM, wound healing, and tissue repair and development showed overlap across the 3 subsets as well as enriched terms that were specific for each subset (Figure 3, B–D). Biological terms associated with the epithelium and endothelium were more heavily enriched in the *Plin2^+^* and *Acta2^+^* subsets compared with the *Col1a1^+^* fibroblasts in the fibrosis-associated DEGs (Figure 3, E and F). Top enriched GO:BP terms for these clusters include regulation of epithelial cell migration and proliferation as well as regulation of endothelial cell migration and proliferation. The top genes associated with these terms that were significantly increased included *Sox9* and *Ppm1f* for epithelial processes and *Prkd2* and *Bmpr2* for endothelial processes (Figure 3, E and F). SSCs associated with apoptosis and lipid storage and production had significant enrichment of GO:BP terms associated with the *Col1a1^+^* and *Plin2^+^* subsets compared with the *Acta2^+^* subset, including regulation of apoptotic processes, regulation of programmed cell death, cellular response to lipid, and regulation of cholesterol storage (Figure 3, G and H). *Fas* and *Bcl2l1* were the top genes with significant upregulation associated with apoptosis (Figure 3G). These results show that we have captured many of the transcriptional changes that have been previously reported in pulmonary fibroblasts during fibrosis development, including changes to ECM organization and the activation of profibrotic fibroblast programming via *Tgfb*, *Notch*, and *Wnt* signaling in all subsets. This suggests that each fibroblast subset is involved in the fibrotic process. Additionally, this suggests that inducible Cre drivers activated during the fibroproliferative process may target multiple profibrotic fibroblast subsets. Apoptotic signaling pathways were enriched in *Col1a1^+^* and *Plin2^+^* fibroblasts, which proceeds the endogenous apoptotic wave that fibroblasts undergo prior to fibrosis resolution beginning 3 weeks after bleomycin (16). Additionally, we identified a subset-specific pathway enrichment associated with epithelial cells in the *Plin2^+^* fibroblast subset and endothelial cells in the *Acta2^+^* fibroblasts.

### Remodeling-associated DEG transcriptional patterns are enriched in Col1a1^+^ and Acta2^+^ fibroblast subsets.

**Figure 4:**
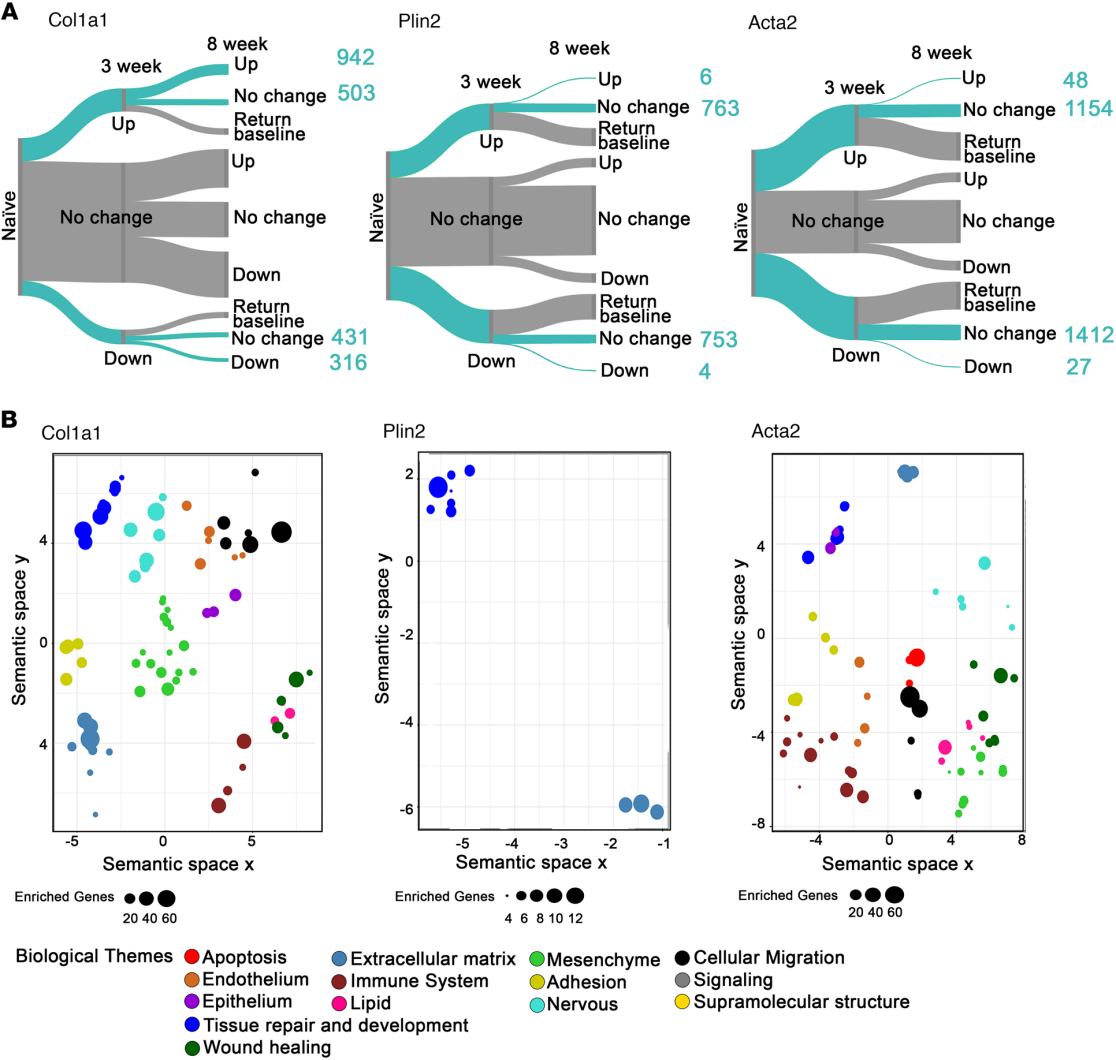
Biological pathways associated with remodeling transcriptional profiles are enriched in Col1a1^+^ and Acta2^+^ subsets. (**A**) Sankey plots for remodeling-associated DEG transcriptional programing in teal, across subsets. Remodeling-associated DEGs are genes significantly altered at 3 weeks versus naive (FDR ≤ 0.05) and either remain altered or continue to be significantly altered (FDR ≤ 0.05) at 8 weeks compared with naive (FDR ≤ 0.05). (**B**) Semantic-similarity clustering of significantly enriched Gene Ontology Biological Process (GO:BP) terms (adjusted *P* ≤ 0.05) derived from highly changed remodeling-associated DEGs (FDR ≤ 0.05, |log_2_FC| ≥ 1) identified 14 semantic similarity clusters (SSCs). Dots are individual GO:BP terms; dot size reflects the number of genes in the term; color indicates SSC.

**Figure 5:**
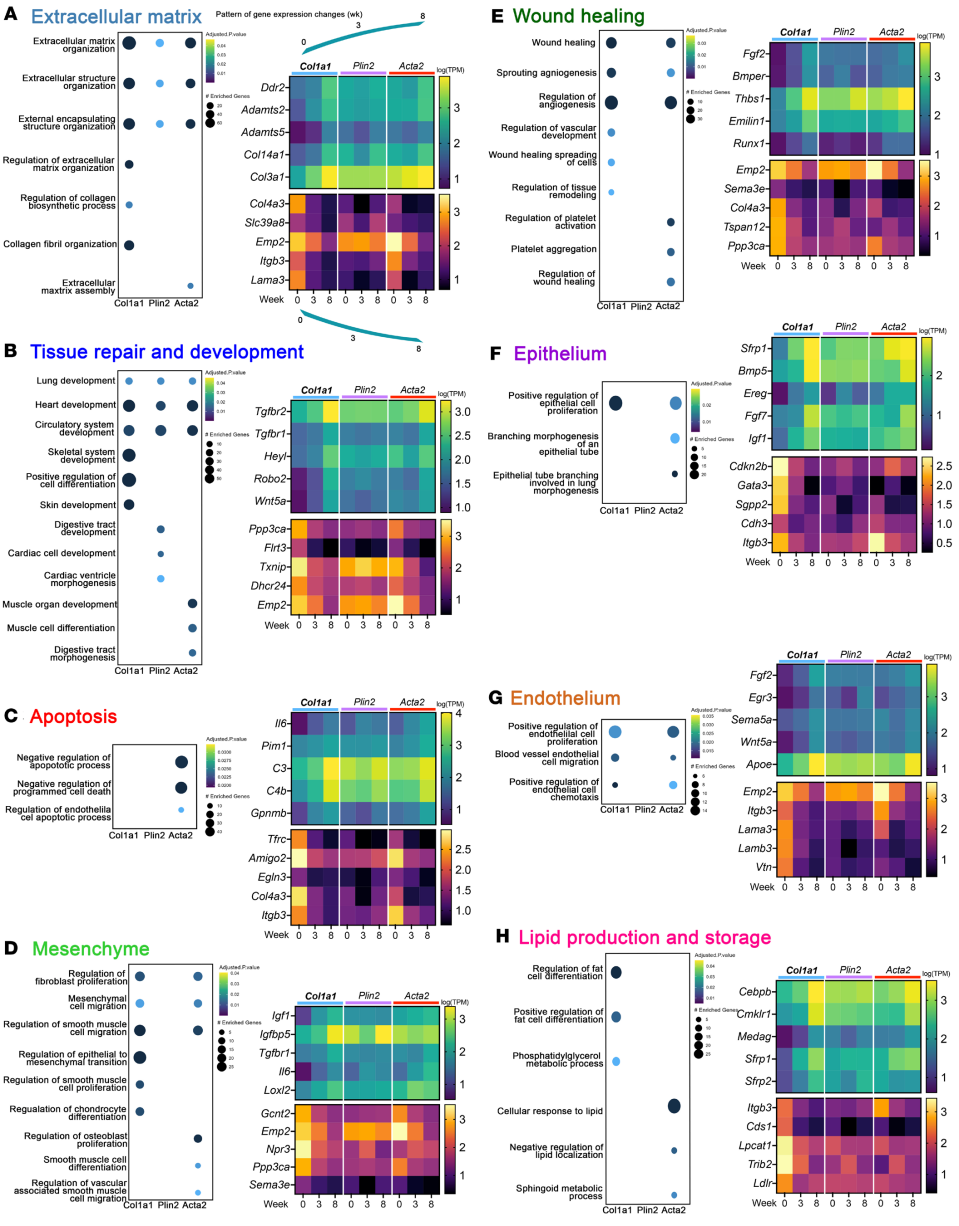
Remodeling-associated transcriptional profile genes are enriched in Col1a1^+^ and Acta2^+^ subsets. (**A**–**H**) Dot plots summarizing representative enriched GO:BP terms and top 5 up- and downregulated genes (log[TPM]) over the time course for selected SSCs based on changes in the *Col1a1^+^* subset: (**A**) extracellular matrix, (**B**) tissue repair and development, (**C**) regulation of apoptosis, (**D**) mesenchymal regulation (**E**) wound-healing, (**F**) epithelial, and (**G**) endothelial regenerative pathways, as well as (**H**) lipid production and storage. Dot size indicates gene count; color indicates adjusted *P* value. Teal schematic over heatmap in **A** indicates expected pattern of gene changes over the time course from the Sankey plot.

Utilizing the same strategy, we examined the fibroblast subsets for SSC enrichment in the DEGs with the remodeling-associated expression profile. These were identified as DEGs that were significantly different between naive and fibrotic lungs (3 weeks) and remained significant at 8 weeks (Figure 4A, teal lines). There were initially 2,192, 1,526, and 2,641 remodeling-associated DEGs for the *Col1a1^+^*, *Plin2^+^*, and *Acta2^+^* subsets, respectively (Figure 4A), that were selected down to those with high expression changes (|logFC| ≥ 1) leaving 1,461, 419, and 1,066 genes for *Col1a1^+^*, *Plin2^+^*, and *Acta2^+^* subsets, respectively, for analysis (Supplemental Figure 5). This resulted in 204, 28, and 249 significantly enriched GO:BP terms in *Col1a1^+^*, *Plin2^+^*, and *Acta2^+^* fibroblast subsets, respectively, consolidated into 14 SSC biological themes that were enriched in at least 1 subset including: apoptosis, endothelium, epithelium, ECM, immune system, lipid production and storage, mesenchyme, adhesion, nervous system, cellular migration, signaling, supramolecular structure, tissue repair and development, and wound healing (Figure 4B). Only 2 SSCs were enriched across all 3 subsets: ECM and tissue repair and development. The top enriched GO:BP terms in these SSCs included ECM organization, lung development, and circulatory system development (Figure 4B and Figure 5, A and B). The most significantly upregulated genes associated with these biological processes across all subsets included *Ddr2*, *Col14a1*, *Col3a1*, *Tgfbr2*, *Tgfbr1*, and *Wnt5a* (Figure 5. A and B). SSCs associated with the mesenchyme, wound healing, the epithelium, endothelium, and lipid production and storage were only enriched within the *Col1a1^+^* and *Acta2^+^* subsets (Figure 4B and Figure 5, D–H). The top enriched GO:BP terms associated with these biological processes include regulation of fibroblast proliferation, wound healing, positive regulation of epithelial cell proliferation, positive regulation of endothelial cell differentiation, regulation of fat cell differentiation, and cellular response to lipid (Figure 5, D–H). Apoptosis pathways were exclusively enriched in the *Acta2^+^* subset with negative regulation of programmed cell death as the most significantly enriched and the most significantly associated DEGs were *Il6* and *Tfrc* (Figure 5C). The most significantly enriched DEGs common to both subsets include *Igf1* and *Gcnt2* for mesenchyme terms, *Fgf2* and *Emp2* for wound healing terms, *Sfrp1* and *Cdkn2b* for epithelial terms, *Fgf2* and *Emp2* for endothelium-associated terms, and *Cebpb* and *Itbg3* for lipid terms (Figure 5, D–H). Interestingly, the *Plin2^+^* fibroblasts had the least amount of DEGs and enriched pathways associated with the remodeling-associated gene expression pattern and compared with the *Col1a1^+^* and *Acta2^+^* subsets. They both had robust and significantly sustained pathway enrichment associated with mesenchyme, ECM, and interactions with other lung cell populations, suggesting a differential role in repair processes between these unique subsets.

### Resolution-associated DEG transcriptional patterns are enriched in the Col1a1^+^ fibroblast subset.

**Figure 6:**
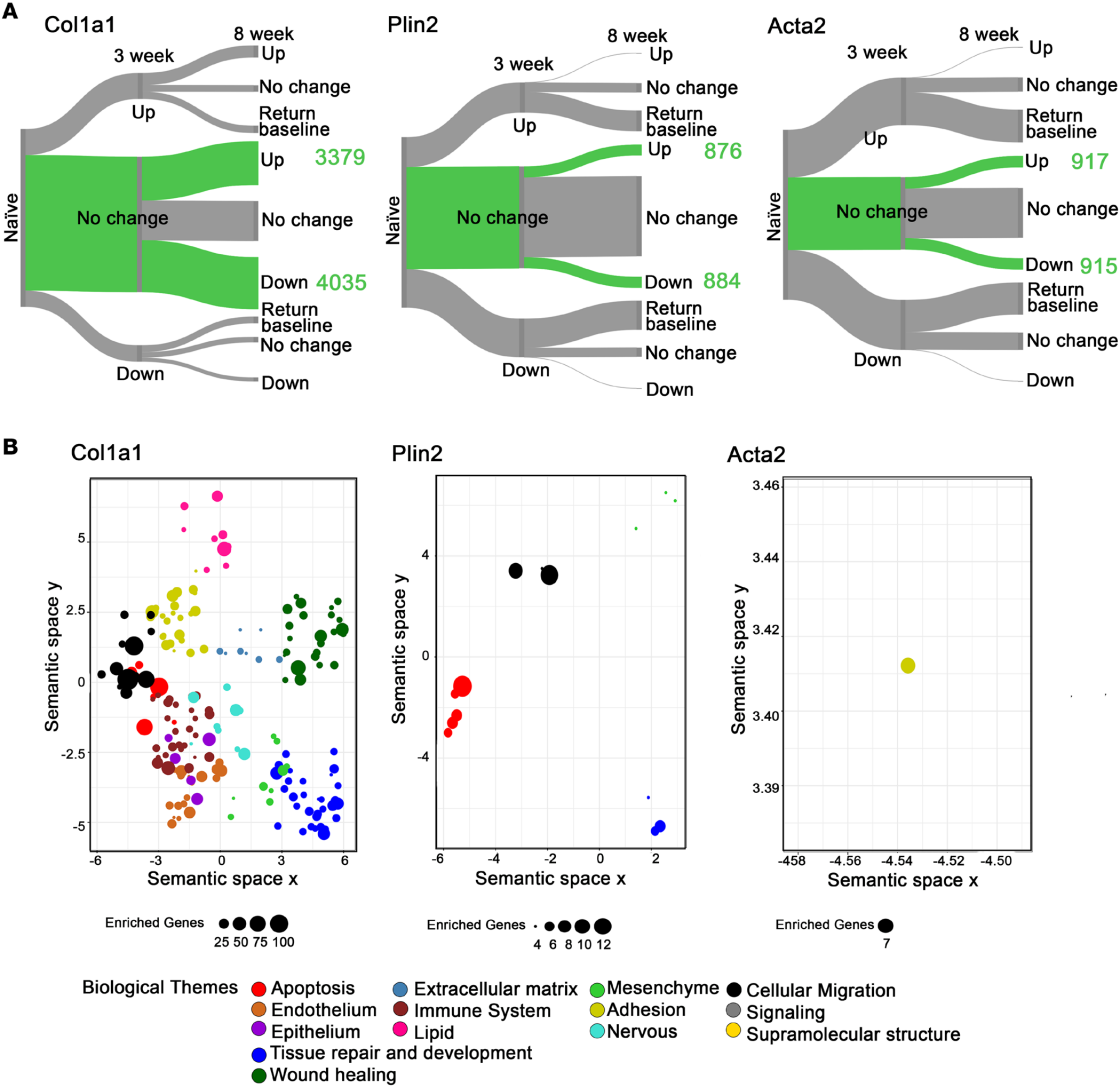
*Col1a1*^+^ fibroblast subset highly express resolution associated transcriptional pathways after fibrosis resolution. (**A**) Sankey plots for resolution-associated DEG transcriptional programing in green, across subsets. Resolution-associated DEGS are genes with significant enrichment only between 3 and 8 weeks (FDR ≤ 0.05). (**B**) Semantic-similarity clustering of significantly enriched Gene Ontology Biological Process (GO:BP) terms (adjusted *P* ≤ 0.05) derived from highly changed resolution-associated DEGs (FDR ≤ 0.05, |log_2_FC| ≥ 1) identified 14 semantic similarity clusters (SSCs). Dots are individual GO:BP terms; dot size reflects the number of genes in the term; color indicates SSC.

**Figure 7:**
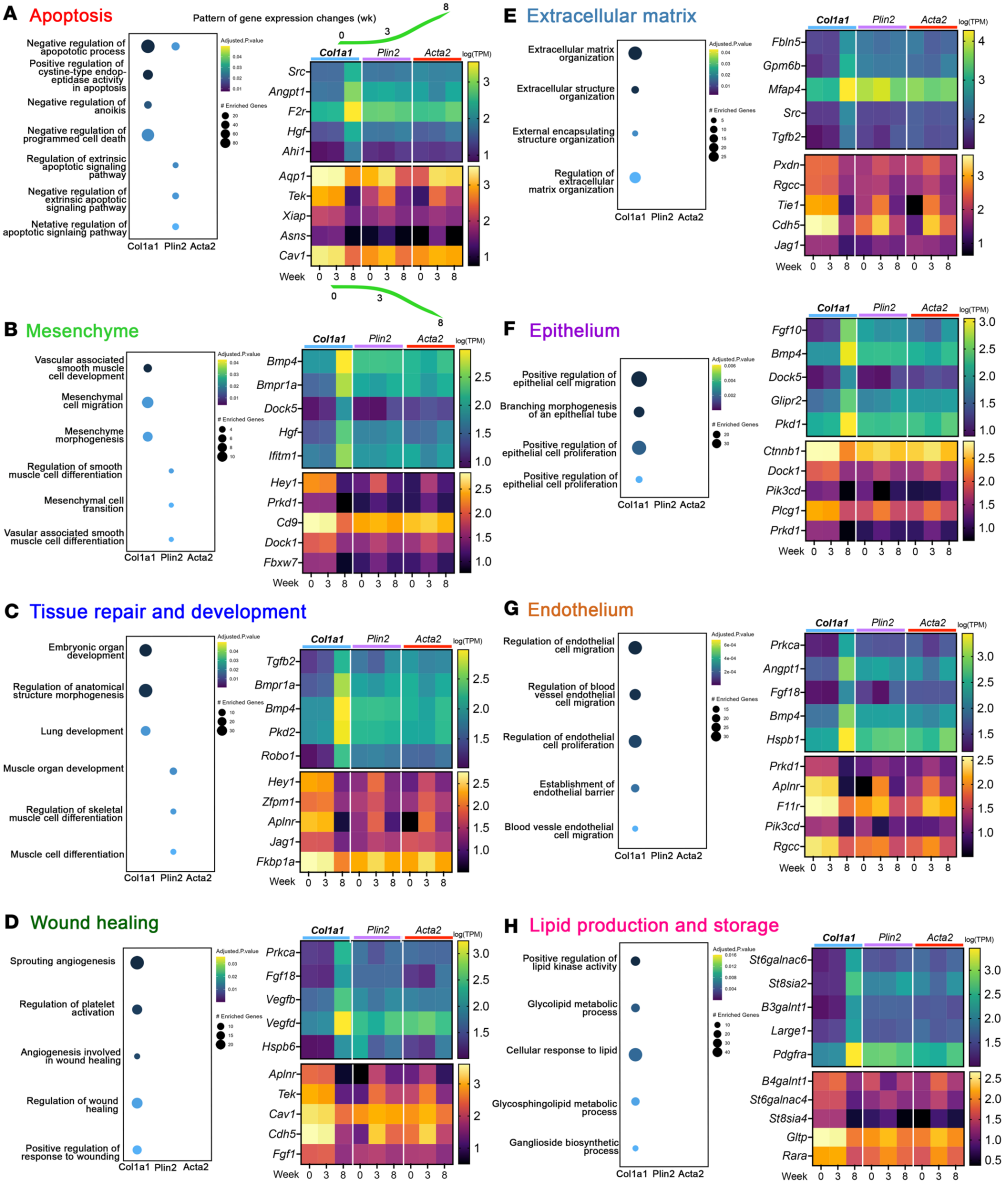
Resolution-associated transcriptional profile genes are selectively enriched in the *Col1a1*^+^ fibroblast subset after fibrosis resolution. (**A**–**H**) Dot-plots summarizing representative enriched GO:BP terms and top 5 up- and down-regulated genes (log(TPM)) over the time course for selected SSCs based on changes in the *Col1a1^+^* subset: (**A**) regulation of apoptosis, (**B**) mesenchymal regulation, (**C**) tissue repair and development, (**D**) wound-healing, (**E**) extracellular matrix, (**F**) epithelial and (**G**) endothelial regenerative pathways, (**H**) lipid production and storage. Dot size = gene count; color = adjusted *P* value. Green schematic over heatmap in **A** indicates expected pattern of gene changes over the time course from the Sankey plot.

Finally, we examined genes that were defined as resolution associated. These were identified as DEGs that were not significantly different between naive and fibrotic lungs but which had significant differential expression between weeks 3 and 8 (Figure 6A). There were 7,414, 1,760, and 1,832 resolution-associated DEGs (FDR ≤ 0.05) for the *Col1a1*, *Plin2*, and *Acta2* subsets, respectively (Figure 6A), with highly changed DEGs (|logFC| ≥ 1) leaving 2,348, 122, and 182 genes for *Col1a1^+^*, *Plin2*^+^, and *Acta2*^+^ subsets respectively, for analysis examined by GO:BP GSEA and SSC (Supplemental Figure 6). This resulted in 386, 38, and 7 significantly enriched GO:BP terms in *Col1a1*^+^, *Plin2*^+^, and *Acta2*^+^ fibroblast subsets, respectively, that were consolidated into 14 SSCs themes identified across 1 or more subset with resolution-associated DEGs including: apoptosis, endothelium, epithelium, ECM, immune system, lipid production and storage, mesenchyme, adhesion, nervous system, cellular migration, signaling, supramolecular structure, tissue repair and development, and wound healing. None were enriched across all 3 subsets (Figure 6B). SSCs enriched for apoptosis, mesenchymal cell interactions, and tissue repair and development in the *Col1a1*^+^ and *Plin2*^+^ subsets (Figure 6B and Figure 7, A–C). The top enrichment terms associated with these clusters included negative regulation of apoptotic processes, vascular smooth muscle cell development, mesenchymal cell migration and cell transition, and embryonic organ and lung development (Figure 7, A–C). The most significant genes associated with apoptosis included *Src* and *Agp1*. Within the mesenchyme cluster, *Bmp4* and *Hgf1* were significantly enriched, and *Tgfb2* and *Hey1* were enriched within the tissue repair and development cluster (Figure 7, A–C). SSCs that were significantly enriched in *Col1a1*^+^ fibroblasts compared with *Plin2*^+^ and *Acta2*^+^ subsets included terms associated with wound healing, ECM, epithelium, endothelium, and lipid production and storage (Figure 7, D–H). GO:BP pathways associated with wound healing were enriched for angiogenesis and platelet activation, with the most significant DEGs being *Fgf18*, *Vegfb*, *Vegfd*, and *Fgf1* (Figure 7D). ECM-associated GO:BP pathways were enriched for ECM matrix organization and ECM structure organization with significant DEGs being: *Mtfap4*, *Fibn5*, *Src*, *Ttgb2*, and *Jag1* (Figure 7E). The *Col1a1*^+^ subset had significant enrichment for GO:BP terms associated with the epithelium and endothelium. These included positive regulation of epithelial cell migration, proliferation and branching morphogenesis of epithelial tubes (significant DEGs: *Fgf10* and *Ctnnb1*), regulation of endothelial cell migration, blood vessel endothelial cell migration, endothelial cell migration, and proliferation and establishment of the endothelial barrier (significant DEGs: *Angpt1*, *Prkca*, and *Prkd1)* (Figure 7, F and G). Finally, the GO:BP terms associated with lipid production and storage that were enriched in the *Col1a1*^+^ fibroblasts were cellular responses to lipid, glycolipid, and glycosphingolipid metabolic processes and regulation of lipid kinase activity and top DEGs, including *St6galnac6* and *B3galnt1*, *Rara*, and *Gltp1* (Figure 7H). Thus, the *Col1a1*^+^ fibroblast subset has a significantly more robust enrichment of SSC, GO:BP terms, and DEGs associated with the fibrosis resolution phase after bleomycin compared with the 2 other subsets. Using the significantly up- and downregulated DEGs from the *Col1a1*^+^ resolution–associated genes at 8 weeks after bleomycin, we enriched for associated transcription factors (TF) and identified 50 significantly upregulated TFs, while 322 were downregulated. The top 10 upregulated TFs were *Jun*, *Runx2*, *Mbd3*, *Cebpd*, *Zfx*, *Gata4*, *Esr1*, *Myc*, *Oling2*, and *Ctcf* (Supplemental Table 1). The top 10 downregulated TFs were *Mecom*, *Irf8*, *Hnf1a*, *Smrt*, *Spi1*, *Ncor*, *Spfi1*, *Tcf7*, *Foxo1*, and *Af4* (Supplemental Table 1). TFs and their associated genes were assessed by GO:BP pathway analysis. Upregulated TFs *Jun*, *Runx2*, and *Myc* were associated with enriched pathways for metabolic processes, lipid modification, fatty acid oxidation, and ER stress. *Mbd3*, *Cebpd*, *Gata4*, *Esr1*, and *Ctcf* were enriched for pathways associated with lung development, cellular differentiation, and regulation of cell communication (Supplemental Table 1). Interestingly, the predominant enriched pathways associated with the downregulated TFs were related to regulation of the immune response, leukocyte activation, cellular response to cytokine signaling, and regulation of lymphocytes, B cells, and myeloid cell differentiation (Supplemental Table 1). Taken together, the enrichment of pathways associated with regeneration of epithelial and endothelial cell populations along with wound repair pathways suggest an active role the *Col1a1*^+^ subset in the regeneration of the fibrotic lung toward a homeostatic structure.

## Discussion

Human and mouse lungs are capable of halting fibrosis and undergoing homeostatic repair processes, as is seen in recovering patients with ARDS and in the single-dose bleomycin mouse model (9–12). Given the central role of fibroblasts in the development of pulmonary fibrosis, we sought to determine the transcriptional profile of fibroblast subsets during fibrosis resolution and lung regeneration. Multiple fibroblast subsets, including those studied herein, have been shown to expand and participate during the development of pulmonary fibrosis. However, very few studies have examined the transcriptional changes in these subsets during disease resolution, and most of these studies have looked at early resolution timepoints — i.e., 5–6 weeks after bleomycin challenge — before the lungs have returned to near-normal histopathology and function (16, 17, 19, 24, 26, 27, 29).

A challenge in defining the functional role of fibroblast subsets during the development and resolution of pulmonary fibrosis lies in their transcriptional and phenotypic plasticity. Pulmonary fibroblasts can differentiate into pathological phenotypes in response to tissue injury, such as fibrosis, and in response to profibrotic signaling pathway activation, with TGF-β being the most well characterized profibrotic fibroblast activator (17, 30–33). Single-cell RNA-seq by our group and others has shown that profibrotic fibroblasts increase transcriptional expression of multiple fibroblast markers often used to identify subsets including *Col1a1*, *Plin2*, *Acta2*, *Runx1*, *Sfrp1*, *Csmd1*, and *Cthrc1* during peak fibrosis (3, 16, 17, 20, 22, 24, 34) and a recently identified *CD248*^+^ proresolving subset during bleomycin resolution at day 35 (3). Thus, to follow and transcriptionally study broad fibroblast subsets that were (a) active during fibrosis, and (b) either remain in the lungs after resolution or were derived from fibrosis-participating daughter cells, we examined TdTm subset–labeled fibroblasts under the *Col1a1*^+^, *Plin2*^+^, and *Acta2*^+^ promoters during fibroproliferation and fibrosis development (days 4–21 after single dose-bleomycin; Supplemental Figures 1 and 7). An important caveat of this labeling approach is that bulk sequencing may obscure specific contributions of subsets contained without a given Cre-defined population, for example the pathogenic *Cthrc1*^+^ fibroblasts that are contained within the broader *Col1a1*^+^ subset (23) (Supplemental Figure 8). This is also relevant to Scube2^+^ alveolar fibroblasts that are PDGFRα^+^ (23) and contained within the *Plin2*^+^ subset (Supplemental Figure 8). Finally, by using a continuous labeling strategy, we expect that the baseline naive population, particularly the *Acta2*^+^ subset, will be different than what is expanded after exposure to a fibrotic stimulus. This makes it important not to overinterpret data comparing naive and fibrotic timepoints; changes may reflect both reprogramming and blurring of lineage definitions when genes such as *Acta2* are upregulated. However, these data accurately capture the populations targeted in experimental strategies when tamoxifen is administered following bleomycin injury. Furthermore, an expected blurring of Cre-defined lineages when fibroblasts are labeled throughout during the fibrotic process makes it even more striking that our study identifies a unique subset-specific enrichment of resolution-associated pathways in *Col1a1*^+^ fibroblasts, which, at the 8-week time point, demonstrate coordinated associations with other mesenchymal populations, ECM components, epithelial cells, and endothelial cells.

Under healthy and homeostatic conditions, *Col1a1*^+^, *Plin2*^+^, and *Acta2*^+^ fibroblast subsets are found in spatially distinct locations of the lung parenchyma with unique transcriptional profiles. *Col1a1* is predominantly expressed in the matrix-producing fibroblasts in the alveolar interstitium that participate in the synthesis and turnover of ECM components (25). *Plin2* is expressed in *Pdgfrα*-expressing alveolar fibroblasts located in close proximity to AT2 cells in the alveolar niche and provide support to AT2 cells through secretion of growth factors and cytokines (IL-6, FGF-7, Wnt) (35). They also transfer phospholipid precursors from alveolar capillary endothelial cells to AT2 cells (36, 37). *Acta2*, while present in myofibroblasts in the developing lungs, is limited to expression in the smooth muscle cells that line airways and large blood vessels in adults (38) and is the dominant subset at our naive time point. During peak fibrosis, the distinct spatial location associated between the 3 subsets was overlapped, and TdTm expression was highly present within the fibrotic lesions for all subsets. Additionally, quantitation of TdTm^+^ cells by flow cytometry showed that all 3 TdTm^+^ subsets underwent significant expansion by 3 weeks after bleomycin. As anticipated, there was an alignment of the global transcriptional profile between all the subsets at 3 weeks after bleomycin and a significant acquisition of a profibrotic profile in all 3 subsets compared with their naive state. This included significant DEG expression of the profibrotic genes *Tgfb1*, collagens, *Smads*, *Pdgfr*, *Wnts*, and *Stats*, and enrichment for profibrotic biological processes including regulation of fibroblast proliferation, ECM production and organization, ECM maintenance and assembly, and regulation of cell matrix adhesion.

After fibrosis resolution, there was a contraction of the TdTm^+^ cell numbers back to naive levels, which is driven by an apoptotic wave beginning at 3 weeks (16, 39) and a reorganization histopathological location of the subsets. However, despite these normalization events as part of the homeostatic repair process, the transcriptional signature of the fibroblast subsets did not completely return to their naive state. This was particularly true of the *Col1a1*^+^ subset, where 69.3% of the DEGs had a significant change in expression during resolution (between 3 and 8 weeks) maintaining a significantly different transcriptional profile. This may be partially attributed to our labeling strategy, where all of the subsets increased *Col1a1* expression (Supplemental Figures 1 and 9), though there was still distinct differentiation of subsets after resolution (Figure 1E). Interestingly, when compared with the Plin2^+^ naive population, there were many similar enriched pathways (Figure 6B and Supplemental Figure 3B). Future linage-tracing experiments might shed light on whether *Col1a1*^+^ fibroblasts revert to a *Plin2*^+^ subset similar to what has been previously shown with *Acta2*^+^ fibroblasts when linage tracing occurred prior to bleomycin injury (24). TF analysis at this time point showed enrichment between TF, resolution-associated genes, and GO:BP pathways that have been linked to pulmonary fibrosis including *Jun*, *Runx2*, *Myc*, *Gata4*, and *Cebpd* and have been identified as having enriched signaling for tissue specific regeneration, metabolic processes, lipid metabolism, ER stress, and TGF-β–dependent signaling. *Gata4* in particular, has been shown to act as a deactivating factor that maintains cells in a quiescent state during homeostasis, and its expression levels are reduced during fibrosis. Its reexpression has been shown to be required for activated fibroblasts to undergo apoptosis or dedifferentiation by inhibiting scarring associated genes including *Snail* in liver and heart fibrosis (40–42).

This resolution-associated transcriptional profile is different from what was observed by Tan et al., who utilized Col1a1;GFP reporter mice to track collagen producing fibroblasts during fibrosis and resolution at day 30 after bleomycin (19). Tan et al. observed that the transcriptional profile of the Col1a1-GFP^+^ fibroblasts was returned toward that of naive cells (similar to what we observed in the *Plin2*^+^ and *Acta2*^+^ subsets) with an elevation in antifibrotic genes and repair pathways (19). However, because this study utilized a GFP reporter, it was not possible to determine if the *Col1a1*^+^ fibroblasts analyzed at day 30 were present throughout the fibrotic process or a new collagen positive population within the lungs. Thus, the strategy of lineage-labeling fibroblast subsets during the active fibroproliferative phase allowed us to address questions about the changes in the transcriptomic profile of fibroblast subsets that not only participated in the fibrosis but that were also participating in fibrosis resolution and repair of the lungs.

Based on the significant transcriptional profile shift in the *Col1a1*^+^ subset at 8 weeks, it suggests that our previous understanding of how *Col1a1*^+^ profibrotic lung fibroblasts contribute to lung repair may not have been fully captured. We observed a significant enrichment of SSCs and associated GO:BP pathways that can be placed into 3 broad themes: (a) mesenchymal cell functions, (b) wound repair processes, and (c) communication/interactions with other cell populations in the lung. Biological processes, such as ECM assembly and maintenance, cell matrix adhesion, mesenchymal cell migration, morphogenesis and differentiation, lung development, regulation of fibrotic proliferation and migration, wound healing, and regulation of tissue remodeling, were all enriched pathways in the differentially expressed resolution-associated genes. However, the biological theme with the most enriched terms were those associated with the regulation of, and communication with, other cells in the alveoli. These include enrichment for multiple terms associated with the positive regulation of epithelial and endothelial cell migration and proliferation, branching morphogenesis of epithelial tubes and new blood vessels, and the establishment of the endothelial barrier. This resolution-associated gene profile suggests that *Col1a1*^+^ fibroblasts may play a role as hubs of coordination for alveolar repair through interactions with many other alveolar structural cells during resolution, which will need to be confirmed. Future mechanistic studies aimed at sorting these unique fibroblast subsets and coculturing with AT2, macrophages, or endothelial cells may help confirm these predictions. Additionally, a recent study has shown Col1a1^+^GFP^+^ fibroblasts can successfully engraft into the lungs 7 days after bleomycin (17). Future studies with the adoptive transfer of Col1a1^+^ fibroblasts from the 8-week resolution time point or “proresolving” fibroblasts (3) into fibrotic lungs may help determine if these transcriptional signatures are functional as hypothesized.

While less studied in the lung during fibrosis resolution, it has been well documented that fibroblasts play essential roles in fibrotic tissue repair and regeneration in other organs. In schistosomiasis-induced and hepatitis B and C liver fibrosis, a significant decrease in liver collagen content and a reversion to normal tissue structure and function have been documented (43–48). Animal models of liver fibrosis including carbon tetrachloride (CCl4) or bile duct ligation–induced pericentral or periportal fibrosis have expansion of profibrotic α-SMA^+^ fibroblasts, primarily originating from hepatic stellate cells, as well as portal fibroblasts, sinusoidal pericytes, and mesothelial cells (49–54). Similar to the lung, the α-SMA^+^ subset has been identified as a key cellular contributor to fibrosis development (49–54). Rapid loss of this population results in a rapid decrease in tissue collagen content, and a reduction in these α-SMA^+^ fibroblast populations correlate strongly with fibrosis regression (55–59). Additionally, activated hepatic cells and portal fibroblasts have been shown to support liver regeneration after fibrosis by secreting growth factors including hepatocyte growth factor (HGF), pleiotrophin, and epimorphin, which stimulate hepatocyte proliferation (60–68). These fibroblasts also regulate immune responses and angiogenesis through increased VEGF and angiopoietin expression. They also facilitate ECM remodeling by producing elevated levels of matrix metalloproteinases (MMP1, MMP2) necessary for collagen breakdown prior to the restoration of normal liver architecture (69–75). Moreover, fibroblasts exhibit phenotypic plasticity, differentiating into progenitor cells through elevated bone morphogenetic protein (BMP) expression to support hepatocyte recovery and tissue repair (76–80). We also see a significant increase in the *Bmp*^+^ differential gene signature in the *Col1a1*^+^ fibroblasts during fibrosis resolution.

Tubulointerstitial fibrosis commonly arises in chronic kidney disease regardless of etiology (81–84). Rodent models of kidney fibrosis, such as unilateral ureteral ligation, confirm that profibrotic fibroblasts originating from resident fibroblasts, epithelial-to-mesenchymal transition, or endothelial-to-mesenchymal transition are central to excessive ECM deposition and increased tissue stiffness (85–91). During fibrosis repair these fibroblasts have been shown to actively promote epithelial proliferation and tubular regeneration through HGF secretion and Wnt/β-catenin signaling activation (92–98). Subsequently, fibroblasts have been shown to return to a homeostatic phenotype, providing critical structural support for tubular regeneration through increased BMP expression (99–102).

In skin-related fibrosis associated with keloid scars and scleroderma, profibrotic papillary and reticular fibroblasts have again been shown to be primary drivers of fibrosis (103–108). Similar to the lung, the resolution of fibrosis correlates with reduced fibroblast numbers after apoptosis and dedifferentiation (109–112). Papillary fibroblasts coordinate wound healing and hair follicle regeneration through the secretion of ECM components such as fibronectin, collagens, and hyaluronic acid, all essential for rapid wound closure and epidermal reepithelialization (103, 113–116). Additionally, fibroblasts increase their production of keratinocyte growth factor (KGF) as part of dermal repair after fibrosis (117).

These examples in other organs suggest that the reparative and cell-interaction transcriptional profiles observed in pulmonary *Col1a1*^+^ fibroblasts are critical for their active role in lung repair during homeostatic fibrosis resolution. We observed significantly increased expression of numerous genes associated with tissue repair specifically involved in epithelial and endothelial regeneration including *Hgf*, *Em1*, *Mmp*s, collagens (4a6,13a1,17a1, 20a1, 23a1), *Vegf*, *Fn*, Fgf7, Fgf10, *Angpt1*, *Prkca*, and *Prkd1* and *Bmps*, all critical for ECM remodeling and reepithelization and endothelization processes necessary to restore alveolar structure and barrier function in the lungs.

Although in some circumstances, including ARDS, the lungs are able to recover and fibrosis resolves, in progressive interstitial lung diseases (ILDs), repair becomes dysregulated and resolution does not occur. Therefore, despite the return to normal lung function and histology in transient pulmonary fibrosis, it is clear that the resolution and reparative transcriptional pathways we observed in the fibroblast subsets in this study do not always become appropriately activated, and we hypothesize this may contribute to progressive fibrotic disease. As we better understand the role of fibroblasts in the repair process itself, we can (a) identify what transcriptional and signaling pathways are absent in ILDs and (b) design novel therapeutic avenues for targeting these fibroblast subsets to reengage their reparative functions.

## Methods

### Sex as a biological variable.

Our study used exclusively male mice as we have shown fibrosis is similar in young male and female mice (1).

### Mouse strains and subset-lineage tagging.

Rosa26R-flox/stop/flox-TdTm (Strain: 007909 B6.Cg-Gt(ROSA)26Sortm9(CAG-TdTm)Hze/J), The Jackson Laboratory) mice were crossed with mice expressing tamoxifen-inducible Cre recombinase driven by *Col1a1* (B6.Cg-Tg(Col1a1-cre/ERT2)1Crm/J; Strain:016241; The Jackson Laboratory). *Plin2* (ADRP-cre/ERT2) (24), and *Acta2* (Acta2-cre/ERT2) (24) were gifted from Stijn De Lange, Mayo Clinic. Recombination was induced by i.p. injection of tamoxifen (0.25 mg/g body weight in corn oil, Sigma Aldrich) at day 4, 8, 12, 16 after bleomycin in 10-week-old male mice to label fibroblast subsets during the fibroproliferative phase with the goal of capturing cells actively increasing these transcripts after bleomycin-indued injury during fibrosis development.

### Fibrosis and assessment of fibrotic lung disease.

Pulmonary fibrosis was initiated by intratracheal instillation of 50 μL of bleomycin (1.5U/kg, Amneal Biosciences, Bridgewater, NJ) to anesthetized mice, as previously described (1). Fibrosis was assessed by measurements of the total collagen in the upper right lung lobe by the hydroxyproline assay, as previously described (1).

### Fluorescence imaging.

TdTm images were acquired from 10 μm fresh-fixed (4% PFA, 20% sucrose in PBS) then OCT frozen (Fisher Scientific) sections mounted with Fluoroshield Mounting Media containing DAPI (Vector Laboratories). Immunofluorescence staining for pro-SPC AT2 cells (Millipore, AB3786, anti-rabbit, 1:500) was followed by incubation with donkey anti–rabbit-A488 secondary (Invitrogen, A31572, 1:100) antibody at 4°C for 1 hour. Images were acquired on a Zeiss Axioplan 2 epi-fluorescence microscope and analyzed with Axiovision software (Zeiss).

### Flow cytometry.

Single-cell suspensions were obtained from perfused, enzymatically dispersed lungs as previously described (16). Cells were stained with fluorescently tagged monoclonal antibodies against CD45 (17-0451-82, Thermo Fisher), CD326/EpCAM (17-5791-80, Thermo Fisher), and CD31 (541814, BD Biosciences at a 1:200 dilution. Fibroblasts were identified as cells expressing TdTm (TdTm^+^) in our lineage negative (CD45^–^, CD326/EpCAM^–^, CD31^–^) population. Cytometry data was acquired with the LSRFortessa (BD Biosciences) and analyzed with FlowJo software (Tree Star).

### Cell sorting and bulk RNA-seq and analysis.

Prior to cell sorting, single cell suspensions were enriched for lineage negative cells by incubating with CD45 (130-052-301, Miltenyi Biotec), CD31 (130-097-418, Miltenyi Biotec), and CD326 (130-105-958, Miltenyi Biotec) MicroBeads and purified off LS columns per manufacturer’s instructions (Miltenyi Biotec, Bergisch Gladbach, Germany). At least 500,000 live (DAPI^–^) lineage negative (CD45^–^,CD31^–^,CD326^–^) cells from 3 mice per line and time point, except for the 8-week *Plin2* group, which has 2 mice, were collected per population on the FACSAria Fusion (BD Biosciences) (Supplemental Figure 9). Purified cells were pelleted and lysed, and RNA was prepared as previously described (16). Libraries were sequenced as barcoded-pooled samples and processed for next-generation sequencing (NovaSeq 6000, Illumina platform). This data set, along with our previously published single-cell data set, has been deposited in the National Center for Biotechnology Information/Gene Expression Omnibus under accession nos. GSE307419 and GSE161648 (16), respectively.

Differential expression analysis was conducted with R (118) package DESeq2 (119), and an FDR less than or equal to 0.05 was used to define significance. GSEA for GO:BP terms (120) was performed using the R ClusterProfiler package (121), and significantly enriched terms were defined as adjusted *P* value less than or equal to 0.05 and had 3 or more genes enriched for the term. Semantic Similarity measurements and clustering were performed using the R GOSemSim (122) package. Sankey plots were generated using SankeyMatic.com. All other plots were generated with the R package ggplot2 (123). For TF analysis, resolution-associated DEGs were separated into up- and downregulated gene sets and were enriched for TF using the ChEA3 database (124) and the R clusterProfiler package.

### Statistics.

Time course data are presented as the mean ± SEM. Data were analyzed using GraphPad Prism software Version 10 (GraphPad). Differences between conditions at specific time points were examined using 2-way ANOVA, with *P* < 0.05 considered to be significant.

### Study approval.

All animal studies were approved by the National Jewish Health IACUC.

### Data availability statement.

All data supporting the findings of this study are available within the paper and the Supporting Data Values file. Sequencing data were deposited into the Gene Expression Ominbus data base under accession no. GSE307419.

## Author contributions

DGF, EFR, NJ, and BPB conducted the experimental work. EFR conceived the project as well as designed and planned experiments. DGF, EFR, NJ, BEV, and DWHR analyzed the data and contributed to writing the manuscript. All authors reviewed the manuscript.

## Conflict of interest

DGF is a current employee of Aetia Scientific Collaborative LLC (ASC), a consulting firm that provides scientific advice to entities including governments, corporations, law firms, and various scientific/professional organizations. This article was prepared and written exclusively by DGF prior to employment at ASC. No outside financial support was provided to any of the authors or their employer for preparing the article.

## Funding support

This work is the result of in part through NIH funding, and is subject to the NIH Public Access Policy. Through acceptance of this federal funding, the NIH has been given a right to make the work publicly available in PubMed Central.

- NIH grants R01HL147860, R01HL149741 and R01HL166250 (EFR).
- NIH grants R01HL156281 and HL140595 (DWHR and EFR).
- NIH grant F31 HL170750-01 (DGF).
- VA Merit Award BX003471 (DWHR).
- TL1 National Center for Advancing Translational Sciences: TR002533 (DGF).
- The Munn Foundation Inc. (EFR).

## Supplementary Material

Supplemental data

Supporting data values

## Version 1 05/05/2026

In-Press Preview

## Version 2 07/08/2026

Electronic publication
