# Supplementary material for: Pulmonary fibroblast subsets demonstrate differentially enriched signaling pathways during fibrosis resolution and repair
Source: JCI Insight. 2026 May 5;11(13):e199660. doi: 10.1172/jci.insight.199660 (PMC13460824; doi:10.1172/jci.insight.199660)
Supplement: Supplementary file 1 [file jciinsight-11-199660-s148.pdf]

SUPPLEMENTAL FIGURES

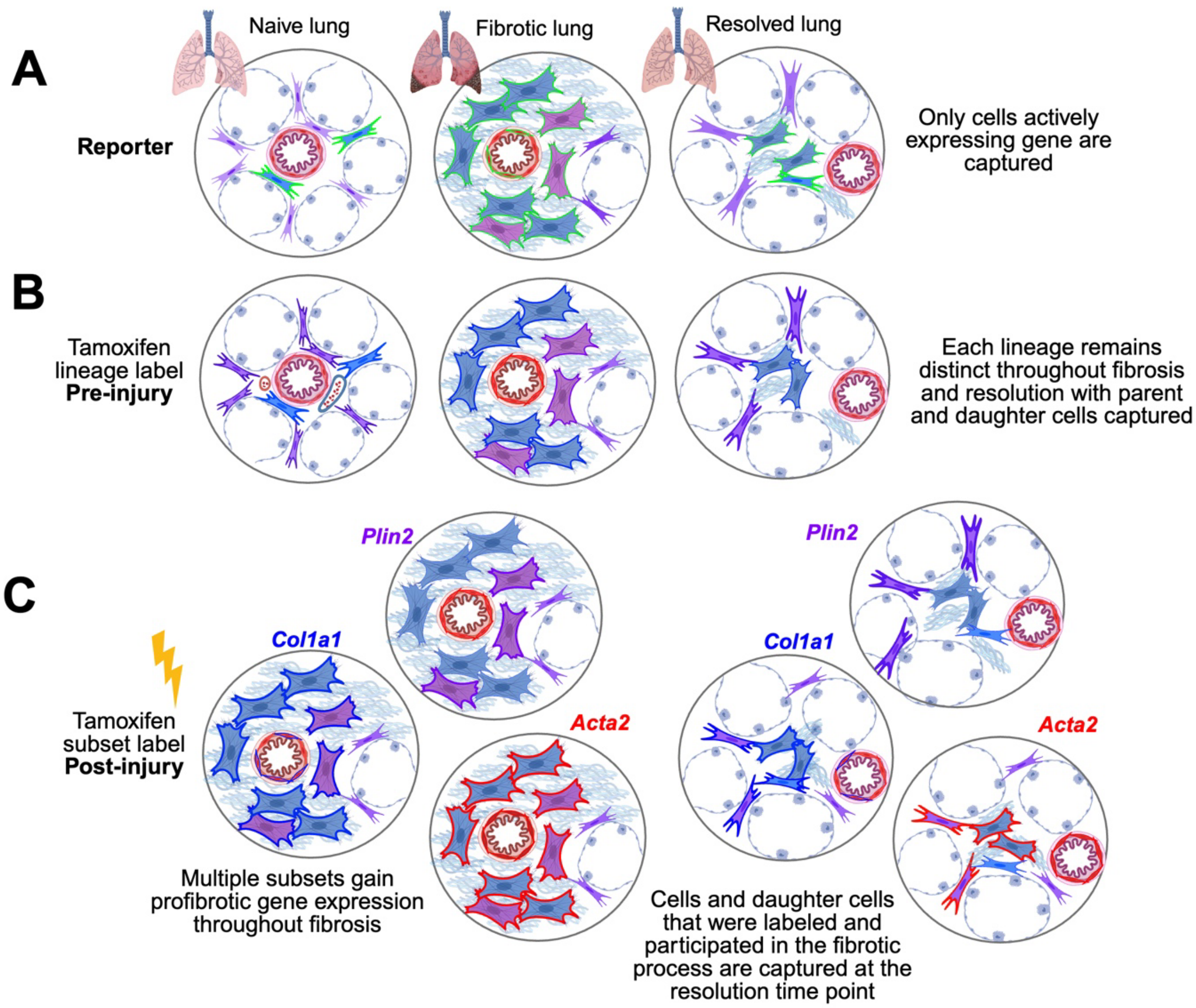

Supplemental Figure 1. Schematics of fibroblast subset labeling strategies at naïve, fibrotic and resolved time points after bleomycin. Individual subsets are colored: *Col1a1* (**blue**), *Plin2* (**purple**) and *Acta* 2 (**red**). A lineage or subset trace is indicated by the bold outline color. (**A**) GFP reporter-based gene expression identification. Different cell lineages may increase or decrease reporter gene expression at different time points during fibrosis or fibrosis resolution. Cells expressing reporter gene outlined in green. (**B**) Inducible Cre-driven lineage labeling when tamoxifen is delivered pre-injury. Lineage is defined in the naïve state and remains throughout fibrosis and fibrosis resolution (cell color and cell outline color match throughout). (**C**) Strategy used in this manuscript: Inducible Cre-driven subset labeling when tamoxifen is delivered post-injury. This strategy labels multiple fibroblast subsets as they gain pro-fibrotic gene expression and participate in fibrosis. It also allows for this actively participating population and their daughter cells to be captured and analyzed during fibrosis resolution. Figure created with Biorender.com.

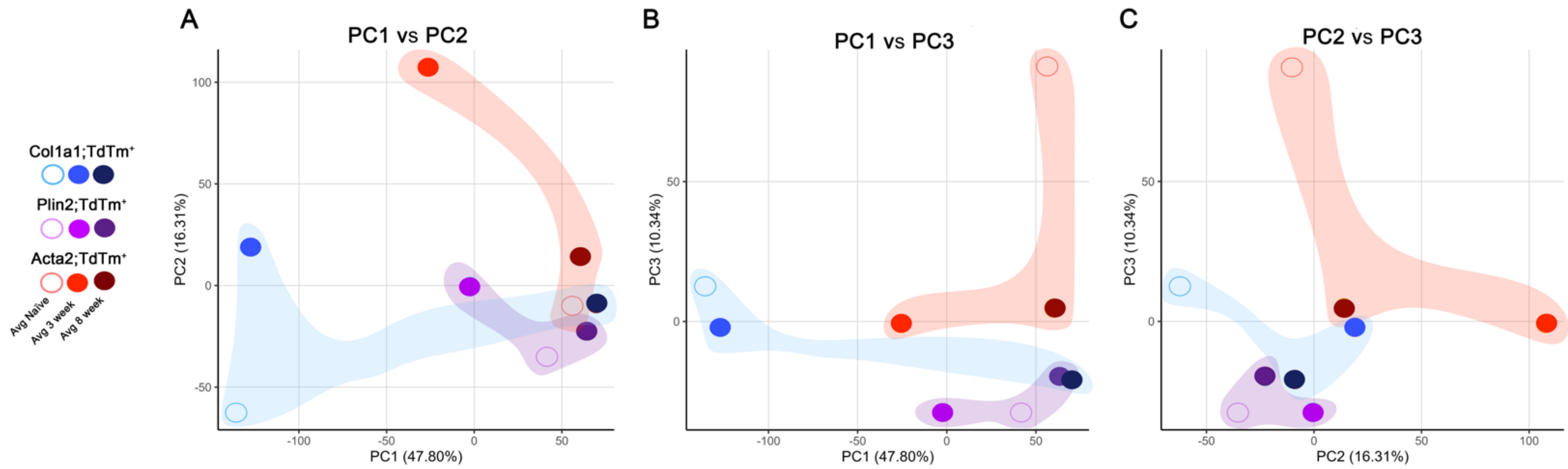

Supplemental Figure 2. Individual 2D PCA plots [graphed as 3D in Figure 1E]. (**A**) PC1 vs PC2, (**B**) PC1 vs PC3 and (**C**) PC2 vs PC3. *Col1a1*+ subsets in blue, *Plin2*+ subsets in purple and *Acta2*+ subsets in red at naïve (open circles) and 3- (bright circles) and 8- weeks (dark circles) post bleomycin.

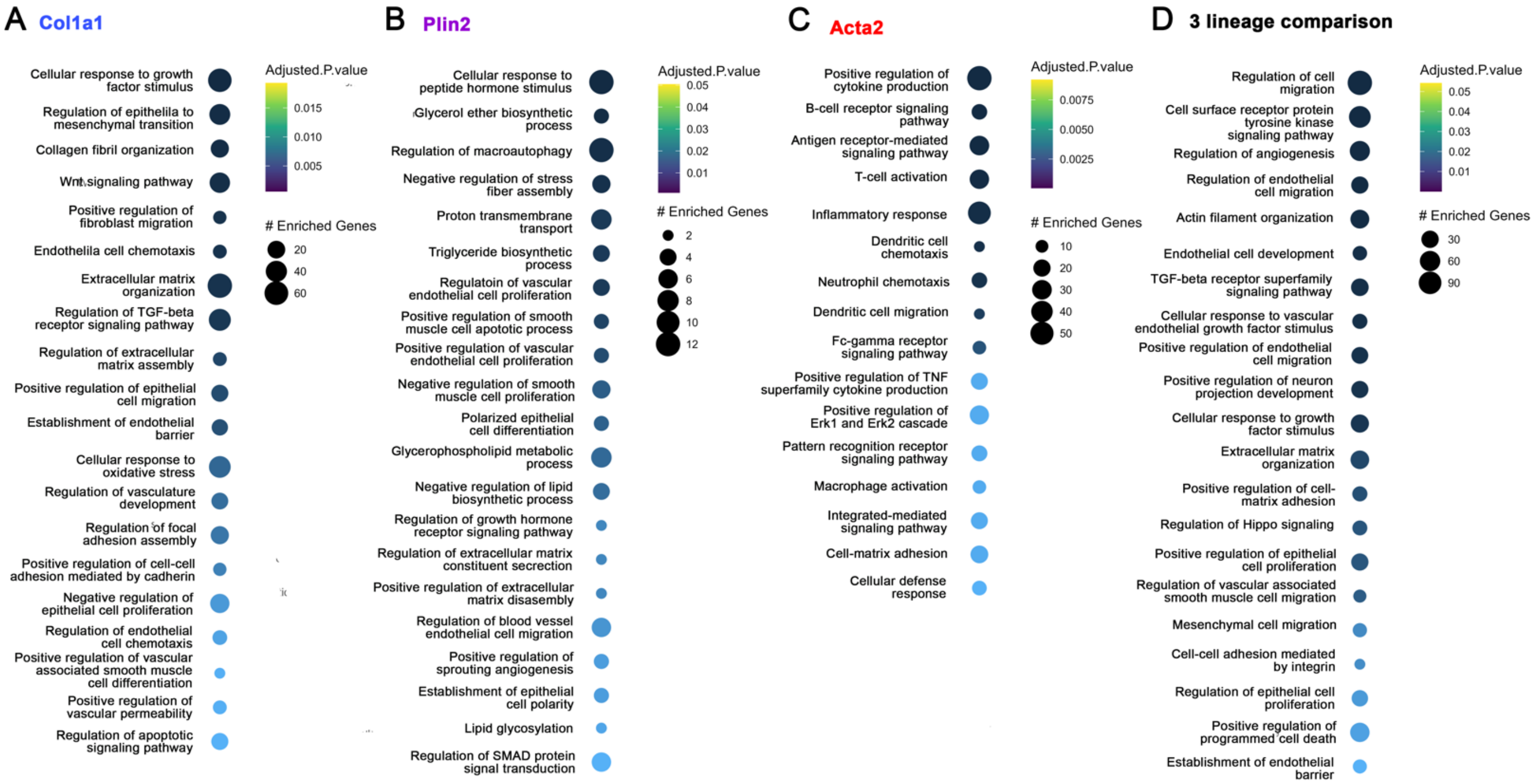

Supplemental Figure 3. Dot-plots summarizing representative enriched GO:BP terms from fibroblast subsets at a naïve baseline. Differentially expressed genes for each subset: (**A**) *Col1a1*+, (**B**) *Acta2*+ and (**C**) *Plin2*+ were analyzed by gene set enrichment analysis for Gene Ontology:Biological Process terms. (**D**) GO:BP terms for genes that were differentially expressed between all three subsets at baseline indicating that all pairwise differences were significant.

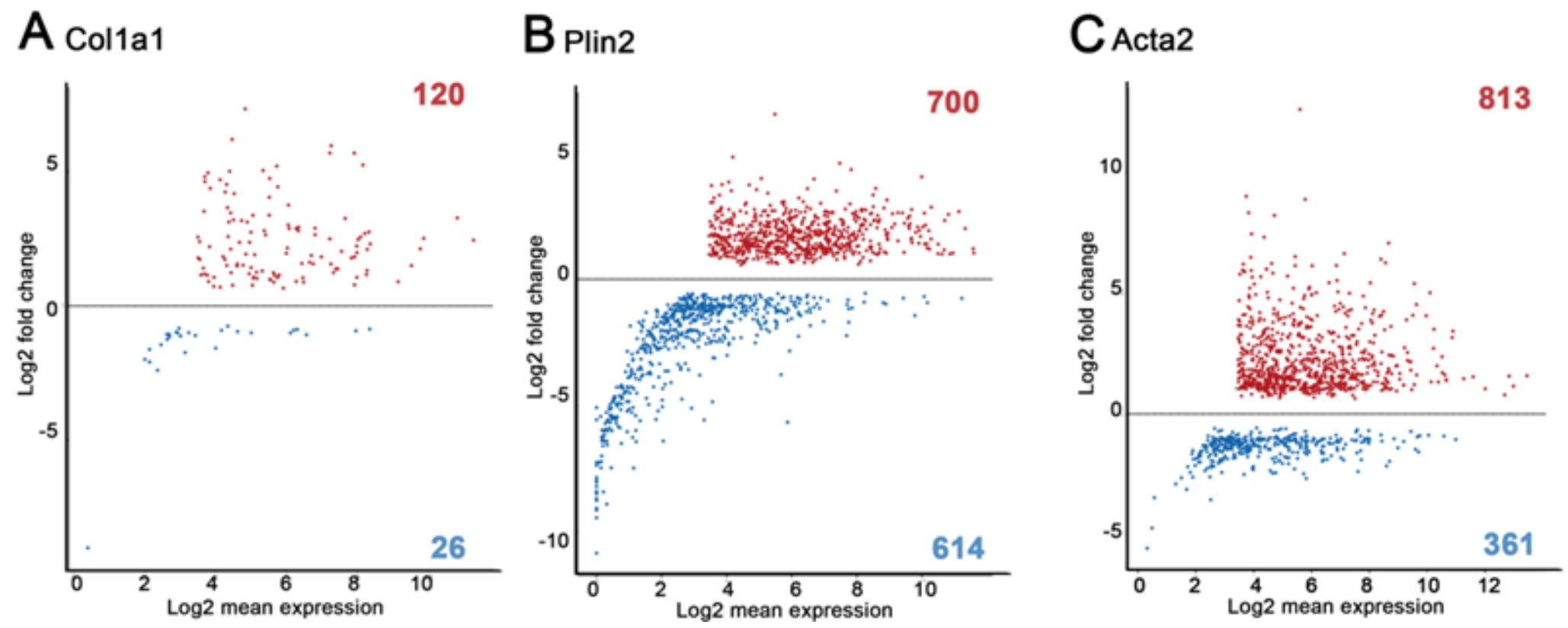

Supplemental Figure 4. MA plots of highly changed fibrosis-associated DEGs (FDR ≤ 0.05, $|log_2FC| \geq 1$) in each subset used for Gene Ontology Enrichment Analysis and Semantic Similarity Clustering. (**A**) *Col1a1+,* (**B**) *Plin2+,* (**C**) *Acta2+* fibroblasts at 3 weeks versus naïve. Red = up-regulated, blue = down-regulated genes.

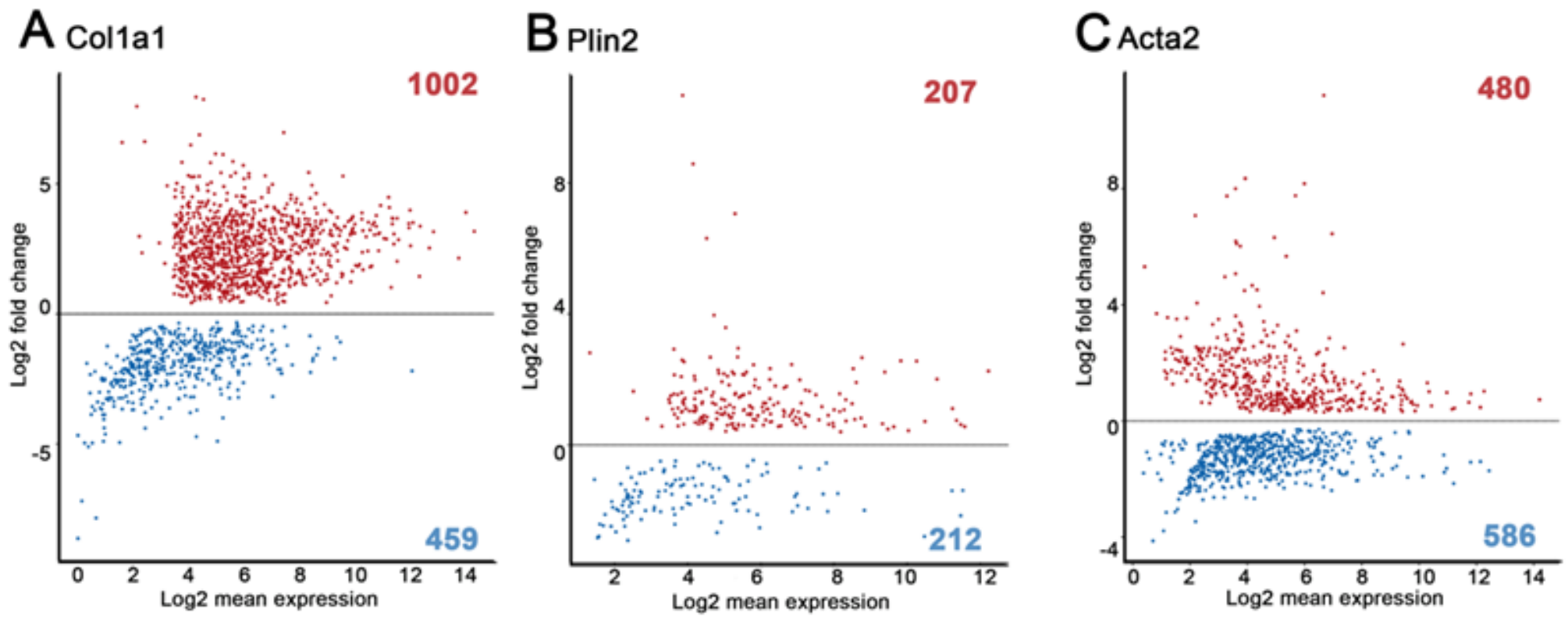

Supplemental Figure 5. MA plots of highly changed remodeling-associated DEGs (FDR ≤ 0.05, $|log_2FC| \geq 1$) in each subset used for Gene Ontology Enrichment Analysis and Semantic Similarity Clustering. (**A**) *Col1a1+,* (**B**) *Plin2+,* (**C**) *Acta2+* fibroblasts at 8 weeks versus naïve. Red = up-regulated, blue = down-regulated genes.

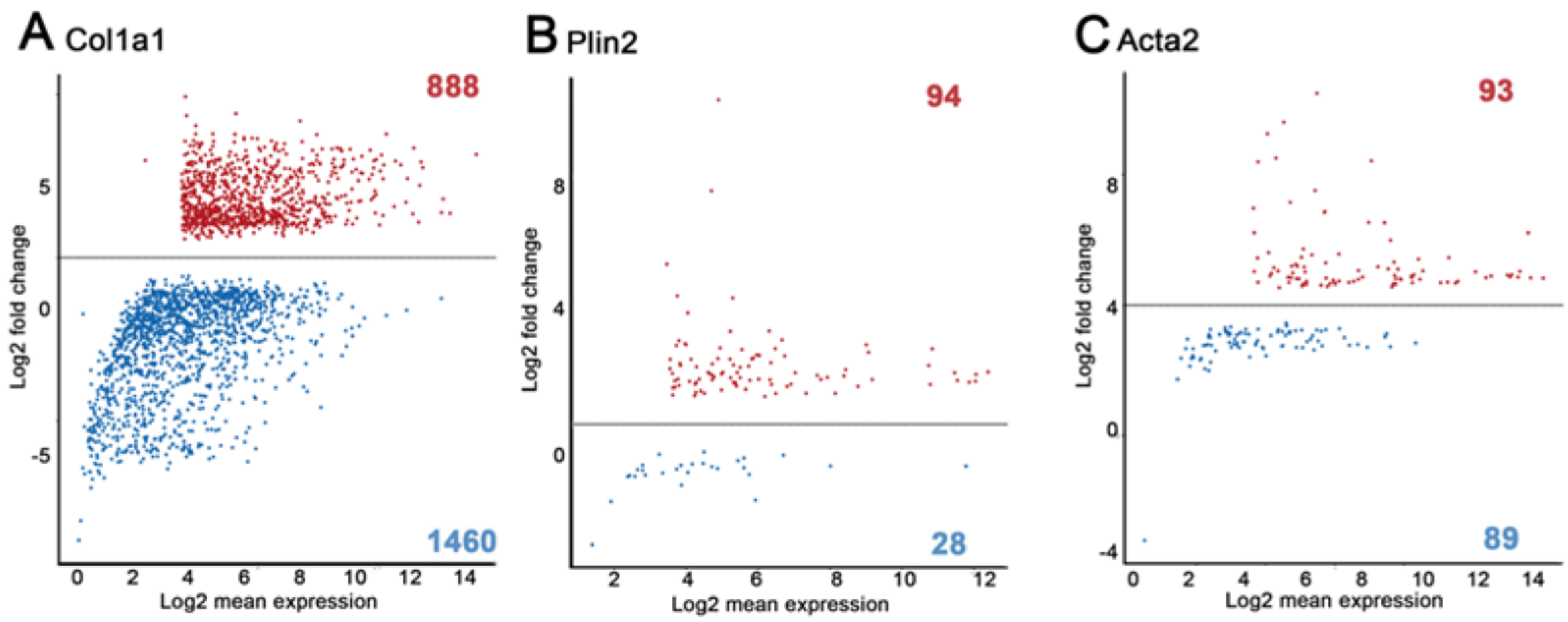

Supplemental Figure 6. MA plots of highly changed resolution-associated DEGs (FDR ≤ 0.05, $|\log_2 FC| \geq 1$) in each subset used for Gene Ontology Enrichment Analysis and Semantic Similarity Clustering. (**A**) *Col1a1+,* (**B**) *Plin2+,* (**C**) *Acta2+* fibroblasts at 8 weeks versus naïve. Red = up-regulated, blue = down-regulated genes.

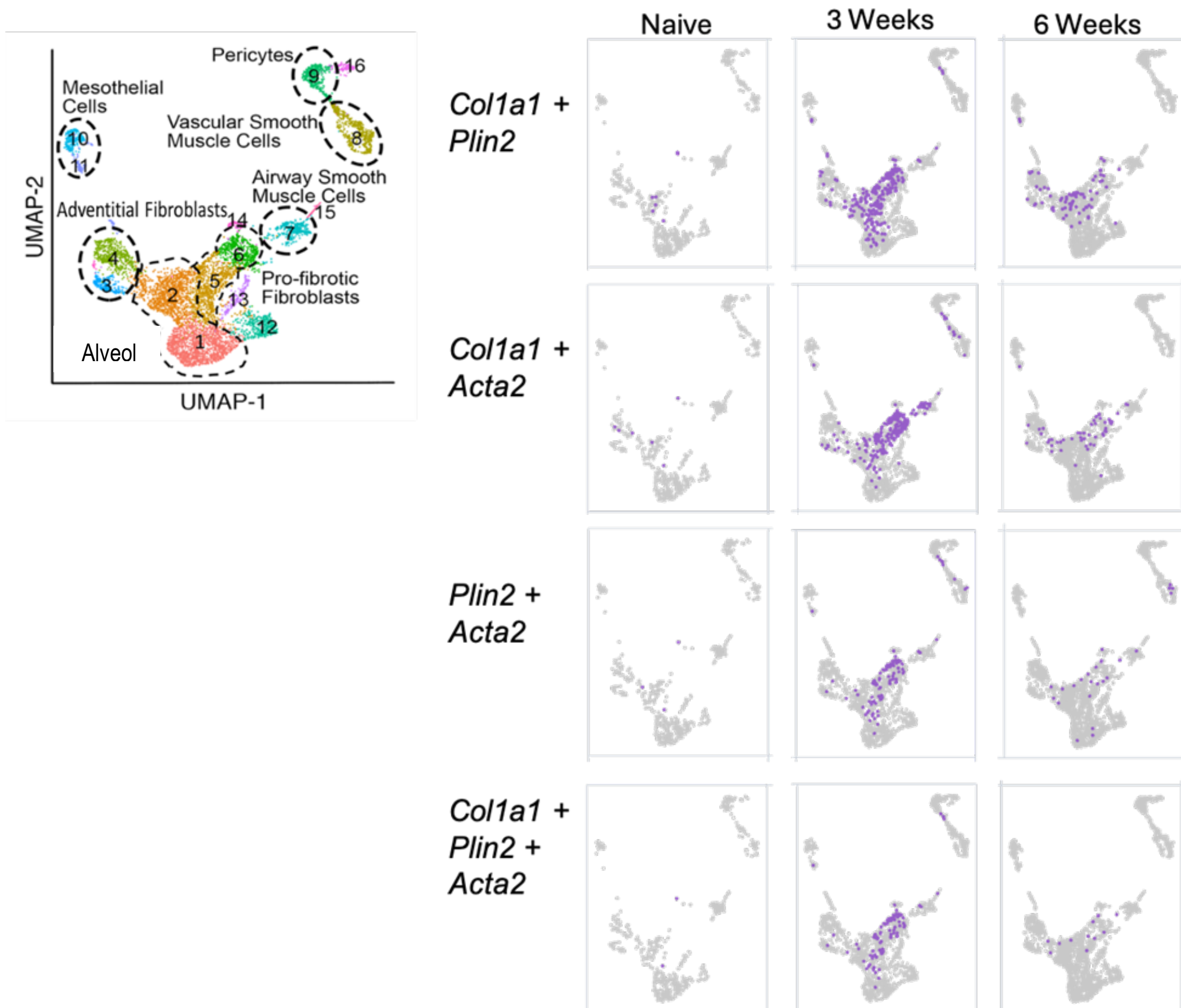

Supplemental Figure 7. Single-cell RNA-seq confirms broad co-expression of overlaid fibroblast-subset markers in the pro-fibrotic fibroblast clusters. UMAP feature-plots generated from our previously published lung scRNA-seq data set (GEO GSE161648) of the pulmonary mesenchyme in three naïve, 3-week, and 6-week post-bleomycin-treated C57BL/6J mice. Overlay of *Col1a1*, *Plin2* and *Acat2* onto clusters 5 and 6 demonstrate the distribution and relative expression changes during fibrosis (3-week) and resolution (6-week) timepoints. Color scale represents log-normalized transcript counts per cell (grey = zero, purple = high).

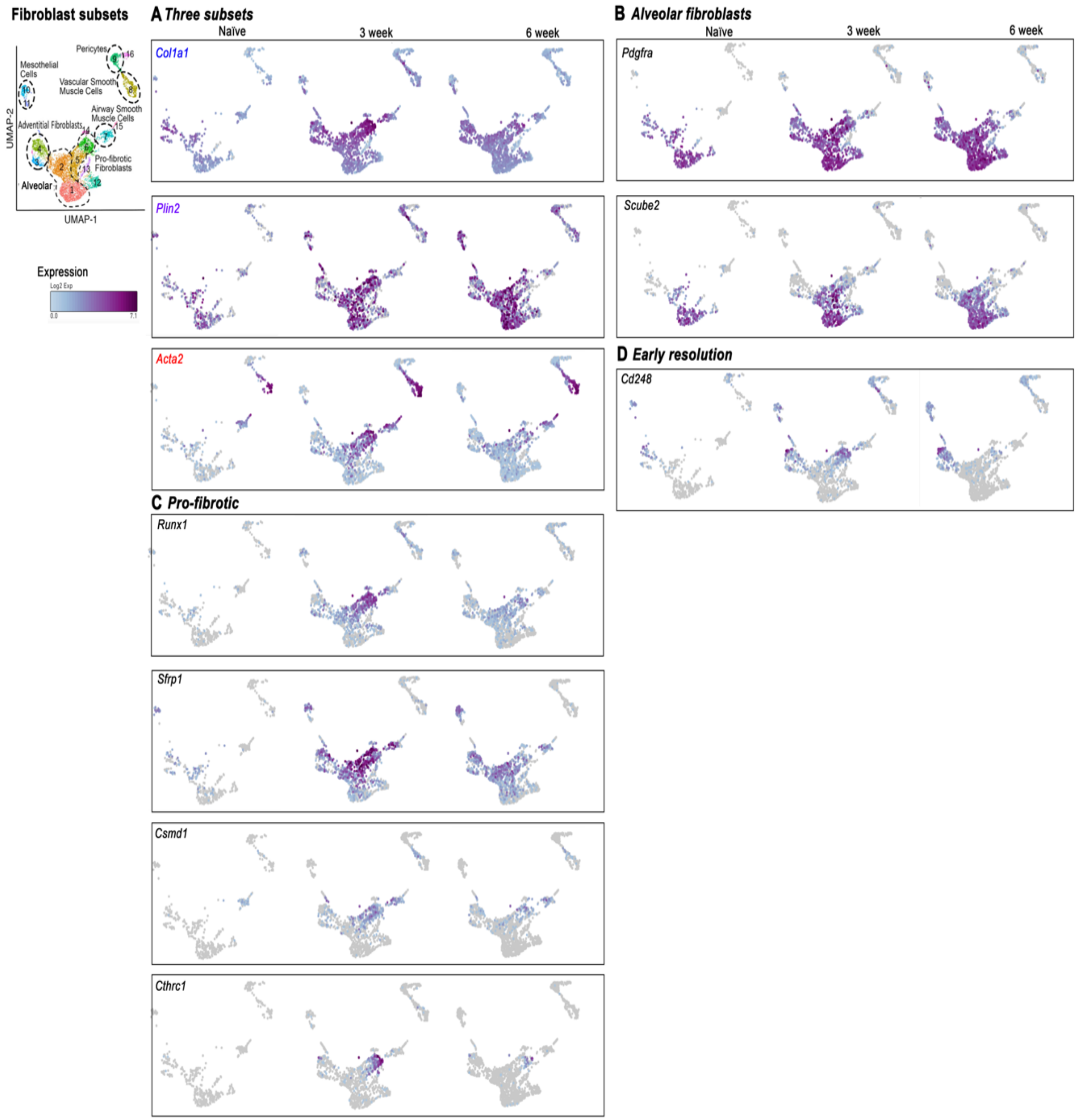

Supplemental Figure 8. Single-cell RNA-seq expression of common fibroblast genes in mesenchymal subsets. UMAP feature-plots generated from our previously published lung scRNA-seq data set (GEO GSE161648) from lung in naïve, 3-week, and 6-week post-bleomycin-treated C57BL/6J mice. (**A**) Expression of our Cre-traced genes Col1a1, Plin2 and Acta2. (**B**) Expression of common alveolar fibroblast genes *Pdgfra* and *Scube2* are present at all time points and broadly expressed in all fibroblast clusters 1-6. (**C**) Expression of common pro-fibrotic genes (*Runx1, Sfrp1, Csmd1, Cthrc1)* show increased expression at week 3 after bleomycin in clusters 5/6. (**D**) Newly identified resolution gene *Cd248* expressed in both adventitial and pro-fibrotic fibroblasts at 3- and 6-weeks post-bleomycin (clusters 3/4 and 5/6). Color scale represents log-normalized transcript counts per cell (grey = zero, purple = high).

A

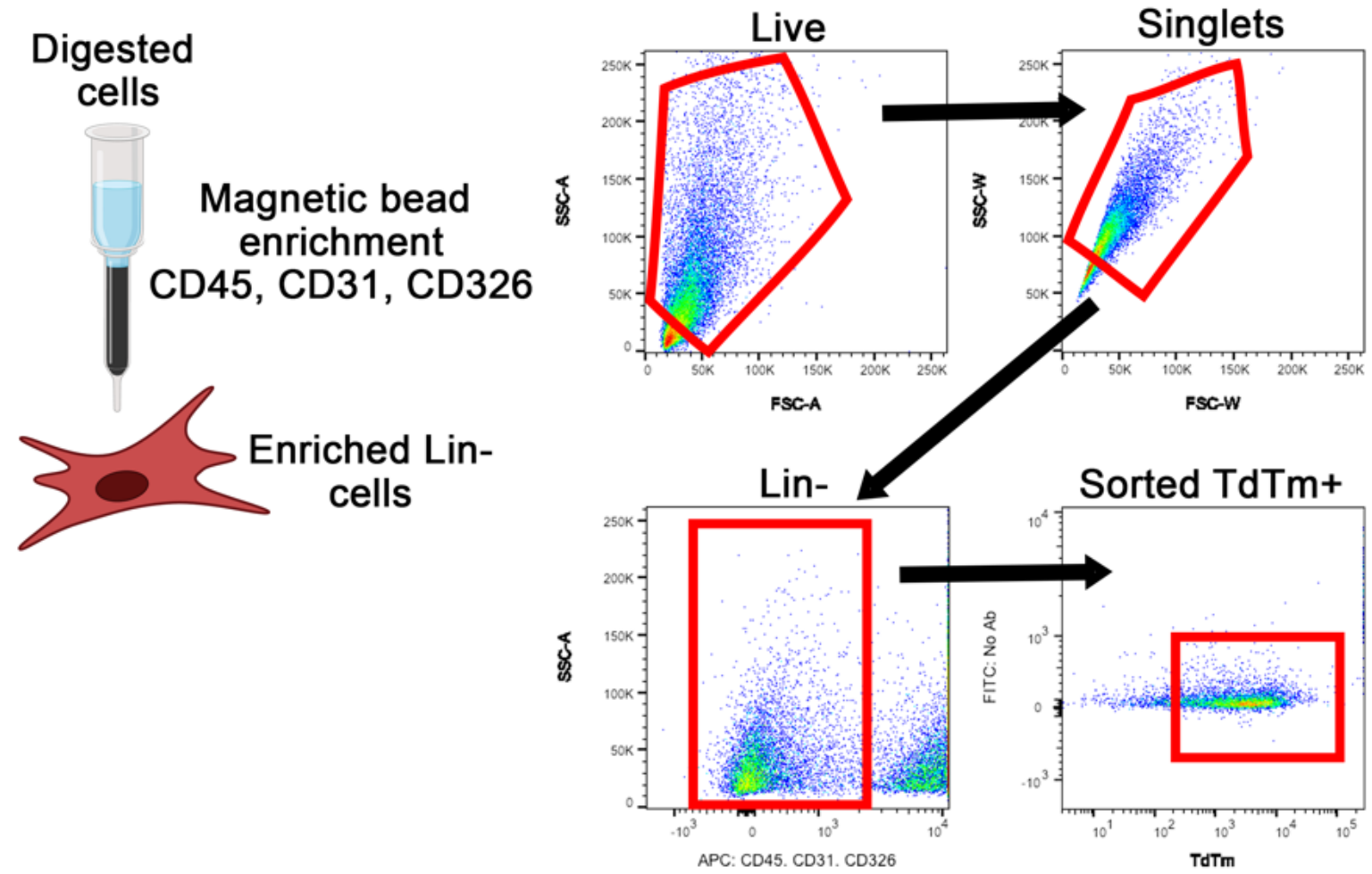

B

Cell Type	Gene	Col1a1 0wk Avg TPM	Col1a1 3wk Avg TPM	Col1a1 8wk Avg TPM	Acta2 0wk Avg TPM	Acta2 3wk Avg TPM	Acta2 8wk Avg TPM	Plin2 0wk Avg TPM	Plin2 3wk Avg TPM	Plin2 8wk Avg TPM
Fibroblast	*Col1a1*	2305.77	2035.66	2749.69	3811.48	11179.84	3901.47	2369.47	2675.37	2418.64
Fibroblast	*Pdgfra*	90.37	107.45	713.65	297.15	239.92	528.84	672.21	708.27	547.20
Fibroblast	*Acta2*	181.40	682.21	1552.39	17941.88	2730.27	5567.85	1016.85	439.36	4782.98
Fibroblast	*Plin2*	81.55	110.52	109.95	60.90	61.88	90.00	663.59	57.45	57.09
Pericyte	*Pdgfrb*	156.60	190.77	280.21	534.86	467.17	355.27	175.36	159.09	211.65
Epithelial	*Epcam*	31.28	27.03	6.12	13.54	2.53	6.51	18.58	0.64	4.07
Endothelial	*Pecam1*	128.91	78.47	21.76	12.02	62.71	38.17	135.39	86.92	93.51
Immune	*Ptprc*	18.66	24.96	3.23	0.98	3.20	4.37	16.21	0.37	8.17

Supplemental Figure 9. Flow cyometery gating strategy and lineage-associated TPM gene expression. (**A**) Enzymatically dispersed lung tissues were enriched on magnetic bead colums to remove CD45+ CD31+ and CD326+ (EpCAM) cells. Enriched Lin- cells were collected and sorted as CD45-CD31-CD326-;TdT+ for bulk RNAsequencing. (**B**) Table of common lineage associated genes and the average transcripts per million (TPM) for each fibroblast subset over time. Genes for non-fibroblast lineages (*Pdgfrb, Epcam, Pecam1, Ptprc)* have low TMP expression as expected.

Transcription Factor	Adjusted *p*-value	Enriched GO Biological Process Pathways	Fold Enrichment	Adjusted *p*-value
**Upregulated**				
Jun	4.95E-14	•Metabolic Processes<br>•Lipid modification<br>•Fatty acid oxidation	1.46<br>3.06<br>4.14	5.17E-19<br>4.99E-02<br>2.61E-02
Runx2	2.57E-10	•Regulation of response to endoplasmic reticulum stress<br>•Fatty acid oxidation<br>•Lipid Oxidation Processes	4.60<br>4.36<br>3.95	9.00E-04<br>5.99E-03<br>2.31E-02
Mbd3	7.38E-10	•Anatomical structure development<br>•Developmental processes<br>•Regulation of cell differentiation	1.24<br>1.29<br>1.56	9.60E-04<br>6.30E-03<br>2.97E-03
Cebpd	2.23E-07	•Anatomical structure morphogenesis<br>•Regulation of transmembrane receptor protein serine/ threonine kinase signaling pathway<br>•Regulation of cellular response to growth factor stimulus	2.09<br>4.28<br>4.09	6.65E-06<br>5.45E-03<br>1.15E-03
Zfx	6.85E-06	•Phosphorus metabolic process<br>•Thioester metabolic process<br>•Acyl-CoA metabolic process	2.1<br>5.2<br>5.2	6.43E-04<br>3.15E-02<br>3.15E-02
Gata4	6.87E-06	•Lung epithelium development<br>•Respiratory tube development<br>•Lung development	10.58<br>4.35<br>4.4	3.69E-02<br>2.11E-02<br>1.81E-02
Esr1	6.59E-05	•Cellular developmental process<br>•Cell differentiation<br>•Positive regulation of cellular process	1.63<br>1.63<br>1.61	2.16E-02<br>2.14E-02<br>1.03E-05
Myc	9.27E-05	•Metabolic process<br>•Protein glycosylation<br>•Golgi vesicle transport	1.47<br>3.13<br>2.62	8.48E-19<br>3.61E-02<br>3.43E-02
Olig2	1.47E-04	•Response to endoplasmic reticulum stress<br>•Endoplasmic reticulum unfolded protein response<br>•Autophagy	5.44<br>9.72<br>3.47	9.36E-06<br>6.99E-04<br>2.76E-02
Ctcf	2.13E-04	•G protein-coupled receptor signaling pathway<br>•Regulation of signaling<br>•Regulation of cell communication	1.28<br>1.45<br>1.43	7.92E-09<br>5.72E-03<br>1.74E-02

Transcription Factor	Adjusted *p*-value	Enriched GO Biological Process Pathways	Fold Enrichment	Adjusted *p*-value
**Downregulated**				
Mecom	3.62E-48	•Regulation of leukocyte activation<br>•Leukocyte cell-cell adhesion<br>•Cell adhesion	4.24<br>11.66<br>2.11	8.21E-20<br>1.87E-12<br>8.42E-03
Irf8	1.27E-43	•Immune system process<br>•Cellular response to cytokine stimulus<br>•Type II interferon-mediated signaling pathway	2.63<br>2.62<br>18.18	5.11E-33<br>7.89E-07<br>3.70E-02
Hnf1a	2.96E-35	•Cellular component organization<br>•Chromatin organization<br>•Chromatin remodeling	1.53<br>2.60<br>2.83	1.47E-17<br>5.55E-14<br>9.11E-13
Smrt	2.96E-35	•Regulation of immune system process<br>•Regulation of leukocyte activation<br>•Regulation of mononuclear cell proliferation	2.85<br>3.68<br>4.47	1.36E-23<br>3.62E-13<br>3.91E-07
Spi1	1.00E-30	•Regulation of leukocyte mediated immunity<br>•Regulation of immune effector process<br>•Negative regulation of B cell receptor signaling pathway	3.7<br>3.13<br>16.3	1.30E-09<br>1.11E-09<br>4.28E-03
Ncor	5.91E-28	•Regulation of immune response<br>•Regulation of innate immune response<br>•Regulation of pattern recognition receptor signaling pathway	3.53<br>4.31<br>4.89	5.72E-23<br>2.48E-16<br>3.64E-06
Spfi1	2.23E-22	•Regulation of lymphocyte activation<br>•Regulation of myeloid cell differentiation<br>•Regulation of immune system process	2.68<br>3.86<br>2.11	6.10E-04<br>3.48E-04<br>1.46E-08
Tcf7	2.37E-22	•Developmental process<br>•Cell differentiation<br>•Regulation of cell development	1.65<br>1.76<br>2.45	9.86E-15<br>1.05E-10<br>1.96E-05
Foxo1	2.40E-22	•Lymphocyte activation<br>•Lymphocyte differentiation<br>•Regulation of B cell activation	4.50<br>5.86<br>5.92	1.25E-17<br>2.02E-16<br>5.01E-06
Af4	6.82E-22	•Immune response-activating cell surface receptor signaling pathway<br>•Regulation of antigen receptor-mediated signaling pathway<br>•Regulation of B cell receptor signaling pathway	3.86<br>5.14<br>9.33	7.16E-08<br>6.18E-04<br>4.86E-02

Supplemental Table 1: Transcription factor enrichment analysis using Resolution-associated DEGs. The top 10 gene-associated transcription factor that were up- or down- regulated were identified using ChEA3 enrichment analysis. Three representative GO:BP pathways for each transcription factor and their significantly associated DEGs genes along with fold change and adj p-value.
